# Insulin promotes the bone formation capability of human dental pulp stem cells through attenuating the IIS/PI3K/AKT/mTOR pathway axis

**DOI:** 10.1186/s13287-024-03843-9

**Published:** 2024-07-29

**Authors:** Lingling E, Yanbo Shan, Yuxi Luo, Lin feng, Yawen Dai, Mingzhu Gao, Yan Lv, Chaoran Zhang, Hongchen Liu, Ning Wen, Rong Zhang

**Affiliations:** 1 Institute of Stomatology & Oral Maxilla Facial Key Laboratory, First Medical Center of Chinese PLA General Hospital, Beijing, China; 2 Department of Temporomandibular Joint, School and Hospital of Stomatology, Guangdong Engineering Research Center of Oral Restoration and Reconstruction & Guangzhou Key Laboratory of Basic and Applied Research of Oral Regenerative Medicine, Guangzhou Medical University, Guangzhou, China

**Keywords:** Human dental pulp stem cells, Insulin, Insulin/insulin-like growth factor-1 signaling (IIS) pathway, PI3K/AKT/mTOR pathway, Bone regeneration

## Abstract

**Background:**

Insulin has been known to regulate bone metabolism, yet its specific molecular mechanisms during the proliferation and osteogenic differentiation of dental pulp stem cells (DPSCs) remain poorly understood. This study aimed to explore the effects of insulin on the bone formation capability of human DPSCs and to elucidate the underlying mechanisms.

**Methods:**

Cell proliferation was assessed using a CCK-8 assay. Cell phenotype was analyzed by flow cytometry. Colony-forming unit-fibroblast ability and multilineage differentiation potential were evaluated using Toluidine blue, Oil red O, Alizarin red, and Alcian blue staining. Gene and protein expressions were quantified by real-time quantitative polymerase chain reaction and Western blotting, respectively. Bone metabolism and biochemical markers were analyzed using electrochemical luminescence and chemical colorimetry. Cell adhesion and growth on nano-hydroxyapatite/collagen (nHAC) were observed with a scanning electron microscope. Bone regeneration was assessed using micro-CT, fluorescent labeling, immunohistochemical and hematoxylin and eosin staining.

**Results:**

Insulin enhanced the proliferation of human DPSCs as well as promoted mineralized matrix formation in a concentration-dependent manner. 10^− 6^ M insulin significantly up-regulated osteogenic differentiation-related genes and proteins markedly increased the secretion of bone metabolism and biochemical markers, and obviously stimulated mineralized matrix formation. However, it also significantly inhibited the expression of genes and proteins of receptors and receptor substrates associated with insulin/insulin-like growth factor-1 signaling (IIS) pathway, obviously reduced the expression of the phosphorylated PI3K and the ratios of the phosphorylated PI3K/total PI3K, and notably increased the expression of the total PI3K, phosphorylated AKT, total AKT and mTOR. The inhibitor LY294002 attenuated the responsiveness of 10^− 6^ M insulin to IIS/PI3K/AKT/mTOR pathway axis, suppressing the promoting effect of insulin on cell proliferation, osteogenic differentiation and bone formation. Implantation of 10^− 6^ M insulin treated DPSCs into the backs of severe combined immunodeficient mice and the rabbit jawbone defects resulted in enhanced bone formation.

**Conclusions:**

Insulin induces insulin resistance in human DPSCs and effectively promotes their proliferation, osteogenic differentiation and bone formation capability through gradually inducing the down-regulation of IIS/PI3K/AKT/mTOR pathway axis under insulin resistant states.

**Supplementary Information:**

The online version contains supplementary material available at 10.1186/s13287-024-03843-9.

## Introduction

The restoration of maxillofacial bone defects caused by cleft lip and palate, congenital anomalies, tooth extractions, tumor resections, or trauma is often compromised by local and systemic pathological conditions [1, 2]. Periodontitis, a chronic inflammatory disease, leads to inflammatory bone resorption, alters alveolar bone structure, and damages tooth-supporting tissues [1], resulting in delayed bone healing at extraction sites of severe periodontitis-affected teeth, with the more complex and unpredictable healing pattern of the alveolar ridge of than that of healthy teeth [3, 4]. Diabetes, characterized by chronic hyperglycemia caused by insufficient insulin secretion or utilization, results in delayed healing of tooth extraction sockets [5] and impaired dental implant osseointegration [2]. These pathological conditions often cause bone defects to fail to heal, leading to chewing difficulties, aesthetic problems, and speech disorders. Therefore, the ability to effectively repair the maxillofacial bone defects in a fully functional and aesthetically pleasing manner is essential to maintain physical and psychological health, which is still a challenge for stomatologists in surgical surgery [6]. Autologous bone transplantation is the gold standard for bone regeneration. However, the limited availability of donor tissue, inevitable additional surgery, and variable bone graft survival limit its practical application in clinical practice [7]. Tissue engineering offers promising alternatives by utilizing stem cells, growth factors, and scaffolds to facilitate the repair of bone defects [8]. Despite considerable advancements, this field still faces numerous challenges, such as the selection of appropriate stem cells, growth factors, and scaffolds, ensuring high implantation rates and the long-term durability of engineered tissues [8].

As a promising cell source, tissue-specific mesenchymal stem cells (MSCs) combined with osteogenic scaffolds and growth factors have been extensively studied both in vitro and in vivo [8]. Human dental pulp stem cells (DPSCs) are one of the important sources of MSCs [9] and capable to differentiate into various cell lineages, including osteogenic, adipogenic, vascular, myogenic and neurogenic lineages [10–12], showing high proliferation rates, superior osteogenic differentiation potential, and robust paracrine and immunomodulatory properties [13–15]. Notably, unlike bone marrow mesenchymal stem cells (BMMSCs) originating from mesoderm cells, DPSCs originate from neural crest cells, possessing the same embryonic origin as maxillofacial bone [15, 16]. BMMSCs have some practical limitations in clinical applications, such as painful collection methods, damage to the donor site, and a reduced proliferation and differentiation ability as the donor ages [13]. While DPSCs can be easily isolated from the pulp tissue of discarded teeth via enzymatic digestion method, with minimal risk of complications [9]. Previous study has reported that DPSCs exhibit better bone regeneration in rat mandibular congenital defects than BMMSCs [13]. These facts provide a distinct advantage in the application of human DPSCs for the maxillofacial bone defects in regenerative medicine.

The division capability and undisputed potential differentiation capability of stem cell play crucial roles in tissue engineering [8]. Insulin, a hormone, known primarily its effects on glucose homeostasis, is essential for various biological functions, such as normal fuel storage, cellular growth, proliferation, and differentiation through correct reception and signaling in the insulin pathway [17, 18]. Increasing evidence suggests that insulin acts as a growth regulator both in vitro and in vivo, stimulating the growth and proliferation of a range of cells, such as somatic cells, osteoblasts, osteoblastic cell lines and stem cells [19–22]. As a standard serum component, insulin is necessary for optimal long-term growth of most cells [19], and is essential for the survival of human embryonic stem cells [21]. Furthermore, insulin is increasingly recognized as an osteogenic inducer. In vivo, insulin treatment has improved the bone abnormalities in type 1 diabetic rodents and facilitated the healing of fractures [23], as well as enhanced the healing of tooth extraction sockets in diabetic rabbits [24]. Mice lacking insulin receptors in osteoblast display low circulating undercarboxylated osteocalcin (OCN) and reduced bone acquisition due to diminished bone formation and a deficiency in osteoblasts numbers [25]. In vitro, insulin has been shown to enhance alkaline phophatase (ALP) activity, type I collagen (COL-1) secretion, *OCN* gene expression, and mineralized nodule formation in MG-63 cells [26], and stimulate the osteogenic differentiation of MC3T3-E1 cells under high glucose conditions [20]. Additionally, the combination of platelet-rich plasma and insulin has induced chondro-/osteogenic differentiation in human adipose-derived stem cells (ASCs) [27] and potentiated bone morphogenetic protein (BMP)-2-induced osteogenic differentiation of rat spinal ligament cells [28]. Insulin is also considered to be a regulator of angiogenesis. Under normal and high glucose conditions, insulin up-regulates the proliferation of human bone marrow-derived endothelial progenitor cells, stimulates the nitric oxide production, reduces the aging of endothelial progenitor cells, and decreases the production of reactive oxygen species, suggesting endothelial protective effects [29]. Restoration of insulin receptor in endothelial progenitor cells rescues vascular function in male mice with insulin receptor haploinsufficiency [30]. Additionally, insulin is involved in regulating inflammatory factors in cells. Topical insulin gel improves wound healing in hyperglycemic mice by modulating the expression of pro- and anti-inflammatory cytokines and promoting the protein expression associated with angiogenesis and insulin signaling pathways [31]. Insulin usually binds to insulin receptor (INSR)/insulin-like growth factor 1 receptor (IGF1R) or INSR-IGF1R complexes through the insulin/insulin-like growth factor-1 signaling (IIS) to exert its functions on cells by modulating downstream signaling pathways of IIS through insulin receptor substrate1/2 (IRS1/IRS2) [32].

The unique properties of human DPSCs and insulin have garnered significant interest in their potential combined use for regenerating maxillofacial bone under various pathological conditions. Notably, previous studies showed that insulin alone not only significantly enhanced the osteogenic differentiation of DPSCs, but also obviously promoted DPSC proliferation [33, 34]. Although retinoic acid (RA) and dexamethasone (Dex) alone also significantly promoted the osteogenic differentiation of stem cells derived from pulp of human exfoliated deciduous teeth (SHED), they markedly inhibited SHED proliferation, and insulin obviously enhanced RA-induced osteogenic differentiation of SHED and attenuated the inhibitory effect of RA on SHED proliferation [35]. BMP comprises a family of proteins of which BMP-2 or BMP-7 is of great importance in the control of bone formation [36, 37]. However, BMP-2 alone not only inhibited DPSC proliferation in a concentration- and time-dependent manner, but also did not induce the osteogenic differentiation of DPSCs in a concentration which caused maximal cell proliferation, and the osteogenic differentiation of DPSCs could only be induced by the combination of BMP-2 and osteoinductive medium [38]. BMP-7 significantly enhanced the osteogenic differentiation of DPSCs stimulated by mineral trioxide aggregate (MTA) under osteoinductive medium, but had no effect on DPSC proliferation [39]. The proliferation potential and differentiation capability of stem cell play crucial roles in tissue engineering [8], and greater proliferation can lead to greater growth of the tissue, which is reflected as an increase in differentiated cell number and not just an increase in the number of stem or progenitor cells [40]. These facts further provide an obvious advantage for the application of insulin to induce the osteogenic differentiation of human DPSCs in maxillofacial bone tissue engineering. Although the effect of insulin on bone formation and the underlying mechanisms has been extensively studied [20, 26–28], the effects of insulin alone treatment on the proliferation, osteogenic differentiation, and bone formation capability of human DPSCs as well as its responsiveness to specific receptor signaling IIS and its downstream PI3K/AKT/mTOR pathway, remain inadequately defined in vitro and in vivo.

The function of tissues and organs depends on cellular behaviors. Scaffolds play a vital role in maintaining high cell density and supporting cellular proliferation and differentiation to simulate physiological conditions. They are also crucial in providing nutritional factors essential for the repair of bone defects [8]. Nano-hydroxyapatite/collagen (nHAC), a ceramic/polymer composite material, has been developed to mimic the nano- to microscale hierarchical microstructure of natural cancellous bone [41]. Owing to its low inflammatory response, good cell compatibility, biodegradability, and its ability to regulate cell growth and differentiation [42, 43], nHAC is considered an ideal scaffold. Our previous research confirmed that rabbit DPSCs adhered, proliferated, and differentiated effectively on nHAC scaffolds [44].

In this study, we aimed to explore the effects of insulin on the proliferation, osteogenic differentiation, and bone formation capability of human DPSCs and to elucidate the underlying mechanisms. We investigated the responsiveness of these cells to the IIS pathway and its downstream PI3K/AKT/mTOR pathway under insulin resistant states. Meanwhile, we assessed the bone formation potential of human DPSCs combined with nHAC and 10^− 6^ M insulin constructs, when implanted subcutaneously into the backs of severe combined immunodeficient mice and into jawbone defects in rabbits over a period of three months.

## Materials and methods

### Isolation and culture of human DPSCs

Human DPSCs were isolated from the dental pulp tissues of thirteen healthy donors aged 19–29 undergoing tooth extraction for therapeutic or orthodontic reasons with no history of pulp inflammation, severe periodontitis, or systemic disease, as our previous method [45]. Briefly, dental pulp tissues were collected from the crown and upper two thirds of the root minced into small fragments, and digested for 1 h at 37 °C using a 1:1 solution of 3 mg/ml Collagenase Type 1 (catalog no. LS004197, Worthington Biochemical Corp., USA) and 4 mg/ml Dispase II (catalog no. D4693, Sigma Aldrich, USA). The resulting cell suspension was cultured in primary culture media composed of alpha-minimum essential medium (α-MEM, Invitrogen, Carlsbad, CA, USA), supplemented with 20% fetal bovine serum (FBS, Invitrogen, Carlsbad, CA, USA), 100 U/ml penicillin and 100 µg/ml streptomycin (Invitrogen, Carlsbad, CA, USA) in a humidified atmosphere of 95% air, 5% CO_2_ at 37 °C. Upon reaching 80 ∼ 90% confluence, cells were passaged, and subsequent cultures were maintained in growth media (GM) composed of α-MEM, 10% FBS, 100 U/ml penicillin and 100 µg/ml streptomycin. DPSCs at passage 5 were utilized for further experiments. The medium was changed every 3 to 4 day. This study was conducted with the approval of the Medical Ethics Committee of the Chinese People’s Liberation Army General Hospital (ethics approval no.: S2022-235-01) and followed institutional guidelines, with informed consent obtained from all participants.

### Cell proliferation assay

Cell proliferation was assessed using the Cell Counting Kit-8 (CCK-8) kit (catalog no. 35,002, Dojindo Molecular Technologies, Inc.). Cells were seeded in a 96-well plate at a density of 2 × 10^3^ cells/well and cultured in 100 µl GM for 24 h to facilitate attachment. Cells were then cultured in 100 µl serum-free GM for 12 h, followed by incubation in 100 µl GM for 1 to 8 days to evaluate the cell growth curve. To examine the effect of insulin on cell proliferation, cells were cultured in 100 µl GM supplemented with varying concentrations of recombinant human insulin (0, 10^− 9^, 10^− 8^, 10^− 7^, 10^− 6^, and 10^− 5^ M; catalog no. I9278, Sigma-Aldrich, Shanghai, China) for 1, 3, 5, and 7 days. Additionally, the effects of insulin and/or the PI3K inhibitor LY294002 on cell proliferation were investigated by culturing cells in 100 µl GM supplemented with 0 M insulin + 0 µM LY294002 (control group), 10^− 6^ M insulin, 10 µM LY294002 (catalog no. L9908, Sigma-Aldrich, Shanghai, China), or 10^− 6^ M insulin + 10 µM LY294002 for 1, 3, 5, and 7 days. Cell proliferation was quantified utilizing the CCk-8 kit.

### Cell phenotype analysis

Flow cytometry was utilized to analyze the phenotype of human DPSCs. Briefly, cells were suspended in 1×phosphate-buffered saline (PBS, Cyagen Biosciences, Inc. USA) at a density of 5 × 10^6^ cells/ml, and stained with specific markers for MSC identification: BD Pharmingen™ PE Mouse anti-Human CD73 (catalog no. 561014), CD105 (catalog no. 560839), CD34 (catalog no. 560941), and BD Pharmingen™ FITC Mouse anti-Human CD90 (catalog no. 561969), CD45 (catalog no. 560976), CD19 (catalog no. 340409), CD11b (catalog no. 562793), and HLA-DR (catalog no. 560944) (BD Biosciences). Antibody binding was assessed utilizing a FACScan flow cytometer (Beckman Coulter), and data were analyzed with FlowJo v10.6.2 (BD Biosciences).

### Toluidine blue staining

The colony-forming unit-fibroblast (CFU-F) ability of cells was evaluated using Toluidine blue staining. Cells were seeded at a density of 100 cells/well in 6-well plates and cultured in 2 ml GM for 10 days. Post-culture, cells were fixed in 10% neutral-buffered formalin for 30 min, stained with 0.1% Toluidine blue solution for 30 min, rinsed twice with 1×PBS and observed under microscope and photographed [42].

The impact of insulin and/or LY294002 on cell proliferation was further assessed by culturing cells in 24-well plates at 1 × 10^5^ cells/ml in 1 ml GM supplemented with 0 M insulin + 0 µM LY294002 (control group), 10^− 6^ M insulin, 10 µM LY294002, or 10^− 6^ M insulin + 10 µM LY294002 for 1, 3, 5, and 7 days. After staining with Toluidine blue, cells were visualized and photographed.

### Oil red O staining

Adipogenic differentiation was determined by Oil Red O staining. Cells were seeded on chamber slides in 24-well plates at a density of 1 × 10^5^ cells/ml and cultured in 1 ml GM. Upon reaching 100% confluence, cells were then induced using OriCell^®^ Human Related Stem Cell Adipogenic Induction Differentiation Kit (catalog no. HUXXC-90031, Cyagen Biosciences Inc, USA) for 21 days. Cells were fixed in 10% neutral-buffered formalin and stained with Oil Red O solution (catalog no. OILR-10001, Cyagen Biosciences Inc, USA). Stained cells were imaged using an inverted light microscope.

### Alkaline phosphatase staining

Alkaline phosphatase (ALP) activity was assessed using the Alkaline Phosphatase Stain Kit (Kaplow’s/Azo Coupling Method) (catalog no.G1480, Solarbio^®^ Life sciences). Briefly, cells were seeded in 24-well plates at a density of 1 × 10^5^ cells/ml and cultured in 1 ml GM. Upon reaching 80 ∼ 90% confluence, cells were treated with varying concentrations of insulin (0, 10^− 9^, 10^− 8^, 10^− 7^, 10^− 6^, and 10^− 5^ M) for 21 days. Parallel groups included cells treated with 0 M insulin + 0 µM LY294002 (control group), 10^− 6^ M insulin, 10 µM LY294002, and 10^− 6^ M insulin + 10 µM LY294002 for 21 days. Post-treatment, cells were fixed in 10% neutral-buffered formalin, stained with ALP Incubation Solution in a dark moist chamber for 15–20 min, washed, and counterstained with Methyl Green Solution for 3–5 min. Cells were rinsed and observed under a microscope and photographed.

### Alizarin red staining

To assess mineralized matrix formation, cells were seeded in 24-well plates at a density of 1 × 10^5^ cells/ml and cultured in 1 ml GM. Upon reaching 80 ∼ 90% confluence, cells were induced using the OriCell^®^ Human Related Stem Cell Osteogenic Induction Differentiation Kit (catalog no. HUXXC-90021, Cyagen Biosciences Inc, USA) for 21 days.

The impact of insulin on mineralized matrix formation was evaluated by treating the cells with varying concentrations of insulin (0, 10^− 9^, 10^− 8^, 10^− 7^, 10^− 6^, and 10^− 5^ M) for 21 days. Additionally, the impact of insulin and/or LY294002 on mineralized matrix formation was assessed by treating the cells with 0 M insulin + 0 µM LY294002 (control group), 10^− 6^ M insulin, 10 µM LY294002, and 10^− 6^ M insulin + 10 µM LY294002 for 21 days.

After treatment, cells were fixed in 10% neutral-buffered formalin for 30 min, stained with Alizarin red solution for 5 min (catalog no. ALIR-10,001, Cyagen Biosciences Inc, USA), observed under a microscope and photographed. To quantify mineralized matrix formation, the Alizarin red stain was eluted from each well with 1 ml of 10% acetic acid solution on the rocking bed for 30 min (the volume ratio of acetic acid and anhydrous ethanol is 8:2), and the absorbance of the eluents was measured at 490 nm using a micro-plate reader [42].

### Alcian blue staining

Chondrogenic differentiation was induced using the OriCell^®^ Bone Marrow Mesenchymal Stem Cells (BMMSCs) Chondrogenic Differentiation Medium Kit (GUXMX-90041, Cyagen Biosciences Inc, USA). Cells were counted after routine digestion, and 3 ∼ 4 × 10^5^ cells were transferred to 15 ml centrifuge tubes, centrifuged at 250 g for 4 min×2 times, and resuspended in 0.5 ml BMMSC chondrogenic differentiation complete medium. This medium consisted of 97 ml OriCell^®^ Basal Medium for Cell Culture, 2 ml OriCell^®^ Supplement For Bone Marrow Mesenchymal Stem Cells Chondrogenic Differentiation I and 1 ml OriCell^®^ Supplement For Bone Marrow Mesenchymal Stem Cells Chondrogenic Differentiation II. The tubes were centrifuged at 150 g for 5 min, keeping the supernatant and the loose cap allowed for gas exchange. The tubes were then incubated at 37 °C and 5% CO_2_. When cell clusters formed (typically within 24 ∼ 48 h), the tube was gently flicked to dislodge the spheroids from the bottom suspending them in the medium. The medium was changed every 2–3 days. After 21 days, the cartilaginous spheroids were fixed in 10% neutral-buffered formalin, embedded in paraffin, sectioned, and stained using Alcian blue to assess chondrogenesis.

### Roche kits analysis

Bone metabolism and biochemical markers were quantified using Roche diagnostic Kits (Roche Diagnostics GmbH). Human DPSCs were seeded in 24-well plates at a density of 1 × 10^5^ cells/ml and cultured in 1 ml GM. Upon reaching 80 ∼ 90% confluence, cells were treated with 1 ml GM supplemented with 0 or 10^− 6^ M insulin for 14 days. Culture media were collected from the wells from day 1 to 7 and from day 7 to 14 for analysis. Total type 1 collagen amino-terminus prolongation peptide (TPINP, catalog no. 47739800) and OCN (catalog no. 49686700) were measured using electrochemical luminescence on a Roche COBAS 8000 system (Roche Diagnostics GmbH, Switzerland). ALP (catalog no. 555262), extracellular calcium (Ca, catalog no. 557586), and extracellular phosphate (P, catalog no. 556864) levels were determined using chemical colorimetry at the Biochemistry Department of Chinese People’s Liberation Army General Hospital, Beijing, China.

### Real-time quantitative polymerase chain reaction (RT-qPCR) assay

RT-qPCR was performed to assess the mRNA expression of the related markers. Cells were seeded in 6-well plate at a density of 1 × 10^5^ cells/ml and cultured in 2 ml GM. Upon reaching 80 ∼ 90% confluence, cells were treated with 2 ml GM supplemented with 0 or 10^− 6^ M insulin for 3 and 7 days. Total RNA was extracted using RNAiso reagent, and cDNA synthesis was conducted using the PrimeScriptTM Reagent Kit. Gene expression was quantified using SYBR^®^ Green Real-time polymerase chain reaction Master mix (catalog no. QPK-201, Toyobo Life Science). The expression levels of *COL-1*, *ALP*, *OCN*, *RUNX2*, *INSR*, *IGF1R*, and *IRS1* were analyzed, with beta-Actin (*β-actin*) serving as the housekeeping gene. Relative gene expression was calculated using the 2-ΔΔCq method, normalized to *β-actin*, and calibrated against the control (cells cultured in GM for 3 days; biological replicates, *n* = 3; technical replicates, *n* = 3). Specific primer sequences were listed in Table 1.

**Table 1:** Primer sequences used for RT-qPCR

Gene	Forward primer (5′-3′)	Reverse primer (5′-3′)
COL-1	AATGTGGTTCGTGACCGTGA	AGCCTTGGTTGGGGTCAATC
ALP	CAACCTCAACCTCAAGCA	GAATGTCTTCTCGTGTAAGTG
OCN	GCAGCGAGGTAGTGAAGA	GAAAGCCGATGTGGTCAG
RUNX2	ATGCGTATTCCCGTAGATCC	GGGCTCACGTCGCTCATTT
INSR	CGACTTCCGAGACCTCTT	TCTACCACCGTCCAACTG
IGF1R	AAGGAATGAAGTCTGGCTCCG	CCGCAGATTTCTCCACTCGT
IRS1	GAGTTCCTTCCGCAGTGTCA	TTGCCACCCCGAGACAAAAT
β-actin	TGACGTGGACATCCGCAAAG	CTGGAAGGTGGACAGCGAGG

### Western blotting analysis

Western blotting analysis was implemented to assess the protein expression of various markers. In short, cells were seeded in 25 cm^2^ culture flasks at a density of 1 × 10^5^ cells/ml and cultured in 5 ml GM. Upon reaching 80 ∼ 90% confluence, cells were treated for 7 days with 5 ml GM supplemented with 0 M insulin + 0 µM LY294002 (control group), 10^− 6^ M insulin, 10 µM LY294002, or 10^− 6^ M insulin + 10 µM LY294002 to determine the effects of insulin and/or LY294002 on protein expression. Proteins were separated by sodium dodecyl sulfate-polyacrylamide gel electrophoresis (SDS-PAGE; Merk Millipore, USA) and transferred to a polyvinylidene fluoride membrane (PVDF; Merk Millipore, USA). Membranes were blocked with 5% non-fat powdered milk (catalog no. D8340, Solarbio) for 1 h. After washing the film with Tris-Glycine Running Buffer (catalog no. T1070, Solarbio), the membranes were probed with primary antibodies including rabbit anti-human glyceraldehyde-3-phosphate dehydrogenase (glyceraldehyde-3-phosphate dehydrogenase GAPDH) (37 kDa) polyclonal antibody (catalog no. AF0911, 1:1000, Affinity Biosciences), rabbit anti-human COL-1 (130–150 kDa) polyclonal antibody (catalog no. AF7001, 1:1000, Affinity Biosciences), rabbit anti-human RUNX2 (57 kDa) polyclonal antibody (catalog no. AF5186, 1:1000, Affinity Biosciences), rabbit anti-human ALP (35 kDa) polyclonal antibody (catalog no. DF12525, 1:1000, Affinity Biosciences), rabbit anti-human OCN (11 kDa) polyclonal antibody (catalog no. DF12303, 1:1000, Affinity Biosciences), rabbit anti-human INSR (95 kDa) polyclonal antibody (catalog no. AF6099, 1:1000, Affinity Biosciences), rabbit anti-human IGF1R (90 kDa) polyclonal antibody (catalog no. AF6123, 1:1000, Affinity Biosciences), rabbit anti-human IRS1 (180 kDa) polyclonal antibody (catalog no. AF6273, 1:1000, Affinity Biosciences), rabbit anti-human PI3K (85 kDa) polyclonal antibody (catalog no. AF6241, 1:1000, Affinity Biosciences), rabbit anti-human p-PI3K (84 kDa) polyclonal antibody (catalog no. AF3242, 1:1000, Affinity Biosciences), rabbit anti-human AKT (55 kDa) polyclonal antibody (catalog no. AF6261, 1:1000, Affinity Biosciences), rabbit anti-human p-AKT (56 kDa) polyclonal antibody (catalog no. AF0016, 1:1000, Affinity Biosciences) and rabbit anti-human mTOR (250–289 kDa) polyclonal antibody (catalog no. AF6308, 1:1000, Affinity Biosciences). After washing in 1×Tris-buffered saline with 0.05% Tween-20, the membranes were probed with a goat anti-rabbit IgG H&L (Horse Radish Peroxidase-conjugated, HRP-conjugated) secondary antibody (catalog no. ab205718, 1:1000, Abcam). Bands were visualized using enhanced chemiluminescence detection reagents (catalog no. SC-2048, Santa Cruz Biotechnology, Inc., USA) and analyzed using Adobe Photoshop CS4 analysis system (Adobe Systems Incorporated, USA). Three independent experiments were performed. Protein expression was quantified relative to GAPDH using the following formula:

$${\mathop{\rm Relative}\nolimits} \,\text{protein}\,{\mathop{\rm expression}\nolimits} = \left( {{\matrix{\text{Mean}\,\text{grayscale} \times \text{area}\,\text{of} \hfill \cr \text{protein}\,{\mathop{\rm expression}\nolimits} \,\text{band} \hfill \cr} \over \matrix{\text{Mean}\,\text{grayscale} \times \text{area}\,\text{of}\, \hfill \cr \text{GAPDH}\,\text{protein}\,{\mathop{\rm expression}\nolimits} \,\text{band} \hfill \cr} }} \right)$$

### Preparation and seeding of nHAC

nHAC scaffolds, procured from Beijing Allgens Medical Science & Technology Co., Ltd. (China) were cut into blocks of 5 mm × 5 mm × 5 mm and 10 mm × 4 mm × 3 mm. These scaffolds were sterilized using cobalt-60 irradiation to ensure aseptic conditions. Cells were seeded onto these scaffolds in 24-well plates at a density of 1 × 10^7^ cells/cm^2^ and cultured in 1 ml GM for 24 h to promote cell adhesion. Subsequently, the seeded scaffolds were cultured in 1 ml GM with or without 10^− 6^ M insulin for 7 days, preparing them for further in vitro and in vivo analyses.

### Scanning electron microscope observation

After seven days of incubation, the constructs (5 mm × 5 mm × 5 mm) of nHAC, DPSCs + nHAC, and DPSCs + nHAC + 10^− 6^ M Insulin were fixed using a mixture of 2% paraformaldehyde and 2.5% glutaraldehyde (Sigma-Aldrich, Shanghai, China). The constructs were thoroughly rinsed in PBS, dehydrated in a graded a series of ethanol solutions, and transitioned through a series of hexamethyldisilazane concentrations for critical point drying. Each construct was mounted on stubs using conducting paste (Nissin EM Co., Ltd., Japan) and sputter-coated with gold to a thickness of several nanometers to enhance electron conductivity. The prepared constructs were then examined under a Hitachi S-520 scanning electron microscope (Hitachi, Tokyo, Japan) to assess the morphology and interaction of cells with the scaffold material.

### Animal surgery and tissue fluorescent labeling

To further explore the osteogenic effects of 10^− 6^ M insulin on human DPSCs, the constructs (5 mm × 5 mm × 5 mm) treated with 1 ml GM supplemented with 0 M insulin + 0 µM LY294002 (control group), 10^− 6^ M insulin, 10 µM LY294002, or 10^− 6^ M insulin + 10 µM LY294002 for 7 days were subcutaneously implanted into the backs of six 6-week-old female severe combined immunodeficient (SCID) mice (18.00 ± 0.53 g) (*n* = 6 per group). The SCID mice were sourced from the Medical Laboratory Animal Center of the Chinese People’s Liberation Army General Hospital, Beijing, China. After the SCID mice were anesthetized using pentobarbital sodium (50 ∼ 60 mg/kg), the skin incision was made on the back of the SCID mice, the skin was separated, and then the constructs were implanted into the subcutaneous skin. Next, the skin was closed with 4 − 0 silk sutures by continuous suture. Each SCID mouse received four constructs (0 M insulin + 0 µM LY294002, 10^− 6^ M insulin, 10 µM LY294002, and 10^− 6^ M insulin + 10 µM LY294002) (Fig. 7B). Post-surgery, the SCID mice (3 per cage) were housed in a controlled environment (20 ∼ 26 °C, 40 ∼ 70% humidity of, 15 air change per hour, 12-hour light/dark cycle) and had access to food and water ad libitum. All procedures were approved by the Animal Care Committee of the Chinese People’s Liberation Army General Hospital (ethics approval no: 2022-X18-48) and followed by institutional guidelines.

Additionally, to assess in situ bone formation, the constructs (10 mm × 4 mm × 3 mm) cultured in 1 ml GM containing 0 (control group) or 10^− 6^ M insulin for 7 days were implanted into jawbone defects of 18 mature female New Zealand white rabbits by random number table method (2.50 ∼ 3.00 kg) (*n* = 9 per group). The rabbits were sourced from the Medical Laboratory Animal Center of the Chinese People’s Liberation Army General Hospital, Beijing, China. Anesthesia was administered using a mixture of 0.5 ml/kg 1:1(V/V) xylazine hydrochloride injection (catalog no. (2015) 070011777, 0.25 mg/kg, HuaMu Animal Health Products Co., Ltd., Jilin, China) and midazolam injection (catalog no. H10980025, 5 mg/kg, Jiangsu Nhwa Pharmaceutical Co., Ltd., China) by intramuscular injection. The rabbit lied on its side on the operating table, the hair was shaved in incisor area of the mandible, and the skin is sterilized with iodized disinfection solution. Along the mouth angle, a 15 mm incision was made on the upper margin of the mandible, and the muscular layer was separated, with fully exposing the mandible above the incisor. The mental nerve was separated and put in the lower margin of the exposed mandible to avoid injury. After the periosteum was separated, a bone defect of 10 mm × 4 mm × 3 mm was made in the mandible above the incisor using a dental ball with a diameter of 1.6 mm, supplemented by copious sterile saline water irrigation. And then the construct (0 M insulin or 10^− 6^ M insulin) was implanted into the jawbone defect, the soft tissue was approximated with interrupted 4 − 0 Vicryl (Ethicon, Inc.) and the skin was closed with 3 − 0 silk sutures by continuous suture. Each rabbit received one construct (Fig. 7C). Post-surgery, the rabbits (1 per cage) were housed individually under standard conditions (16 ∼ 26 °C, 40 ∼ 70% humidity, 8 air changes per hour, 12-hour light/dark cycle) and had access to food and water ad libitum. Eleven weeks post-implantation, bone formation was evaluated using calcein labeling (10 mg/kg of body weight, Sigma-Aldrich). All procedures were approved by the Animal Care Committee of the Chinese People’s Liberation Army General Hospital (ethics approval no: 2022-X18-48) and followed by institutional guidelines.

### Micro-CT detection

Three months post-surgery, six of the eighteen rabbits were euthanized using an overdose of anesthesia (*n* = 3 per group). The harvested jawbone specimens were fixed in 10% neutral-buffered formalin, embedded in methyl methacrylate, and scanned using the Quantum GX µCT System at 70 kV, and 114 µA, achieving a resolution of 4.5 μm accuracy. The Quantum GX µCT Workstation software was utilized for the three-dimensional reconstruction of the jawbone defects. The bone mineral density (BMD), bone volume/total volume (BV/TV), and cortical bone volume/total volume (CV/TV) were calculated to assess bone regeneration.

### Confocal laser scanning microscopy observation

Following micro-CT evaluation, jawbone specimens were prepared for confocal microscopy without demineralization (*n* = 3 per group). All specimens were trimmed using waterproof polishing paper without demineralization, sectioned to a thickness of 5 μm, and observed using confocal laser scanning microscopy (Carl Zeiss 510 META, German). Calcein, indicative of mineral deposition, was visualized in green under an argon laser excitation at 488 nm.

### Immunohistochemical staining

Three months post-surgery, six SCID mice and twelve rabbits were euthanized using an overdose of anesthesia (*n* = 6 per group). Specimens were collected, fixed in 10% neutral-buffered formalin, thoroughly washed, and then decalcified with 15% ethylene diamine tetraacetic acid, with the decalcifying solution refreshed one to two times per week. The decalcification process lasted approximately one week for mouse specimens and three months for rabbit specimens. Subsequently, specimens were embedded in paraffin and sectioned to a thickness of 5 μm. The sections underwent immunohistochemical staining as follows: sections were deparaffinized in xylene and rehydrated through graded alcohols to water. Endogenous peroxidase activity was blocked using a commercial peroxidase blocker (catalog no. PV-9001, Zhongshan Jinqiao Biotechnology Co., LTD, China) at room temperature for 10 min. After washing with 1 × PBS, sections were incubated overnight at 4 °C with a primary rabbit polyclonal antibody against human OCN (1:50, catalog no. ab198228, Abcam, UK). Following primary antibody incubation, sections were washed and incubated with a reaction enhancement solution (catalog no. PV-9001, Zhongshan Jinqiao Biotechnology Co., LTD, China) at 37 °C for 20 min. After another wash, sections were treated with enzyme-labeled goat anti-rabbit IgG polymer (catalog no. PV-9001, Zhongshan Jinqiao Biotechnology Co., LTD, China) at 37 °C for 20 min. Following final washes, chromogenic detection was performed using a diaminobenzidine (DAB) solution (catalog no. ZLI-9018, Zhongshan Jinqiao Biotechnology Co., LTD, China). Sections were counterstained with hematoxylin to visualize nuclei. Stained sections were examined and photographed using an inverted light microscope. This detailed protocol ensures consistent and reproducible staining, critical for the reliable visualization of protein expression in tissue samples.

### Hematoxylin and eosin staining

Tissue sections were stained using hematoxylin and eosin (H&E) to assess morphological details (*n* = 6 per group). Following deparaffinization and rehydration, sections were stained with hematoxylin for 5 min, followed by eosin staining. After staining, sections were dehydrated in ascending ethanol concentrations and mounted. For morphometric analysis, one section was selected every 5 sections, and five consecutive sections per specimen were obtained to evaluate the percentage of bone formation area. Five fields of view were selected for each section per specimen at 100 × magnification under an inverted light microscope and were calculated using a Leica Qwin v3.2 image analysis system (Leica Microsystems Inc., Germany). Total scores per section were calculated and averaged for all sections to obtain an overall score for each specimen. Data were then averaged across 6 specimens within each group.

### Statistical analysis

Data were presented as mean ± standard deviation (SD). Statistical analyses were performed using SPSS19.0 (IBM Corp.). Differences between the two groups were assessed using Student’s t-test, while multiple group comparisons were conducted via one-way ANOVA with LSD’s multiple comparison test for homogeneous variances and Tamhane’s T2 test for data with non-normal distribution or unequal variances. Significance levels were set at *p < 0.05, **p < 0.01, and ***p < 0.001.

## Results

### Isolation, culture, and characterization of human DPSCs

**Fig. 1:**
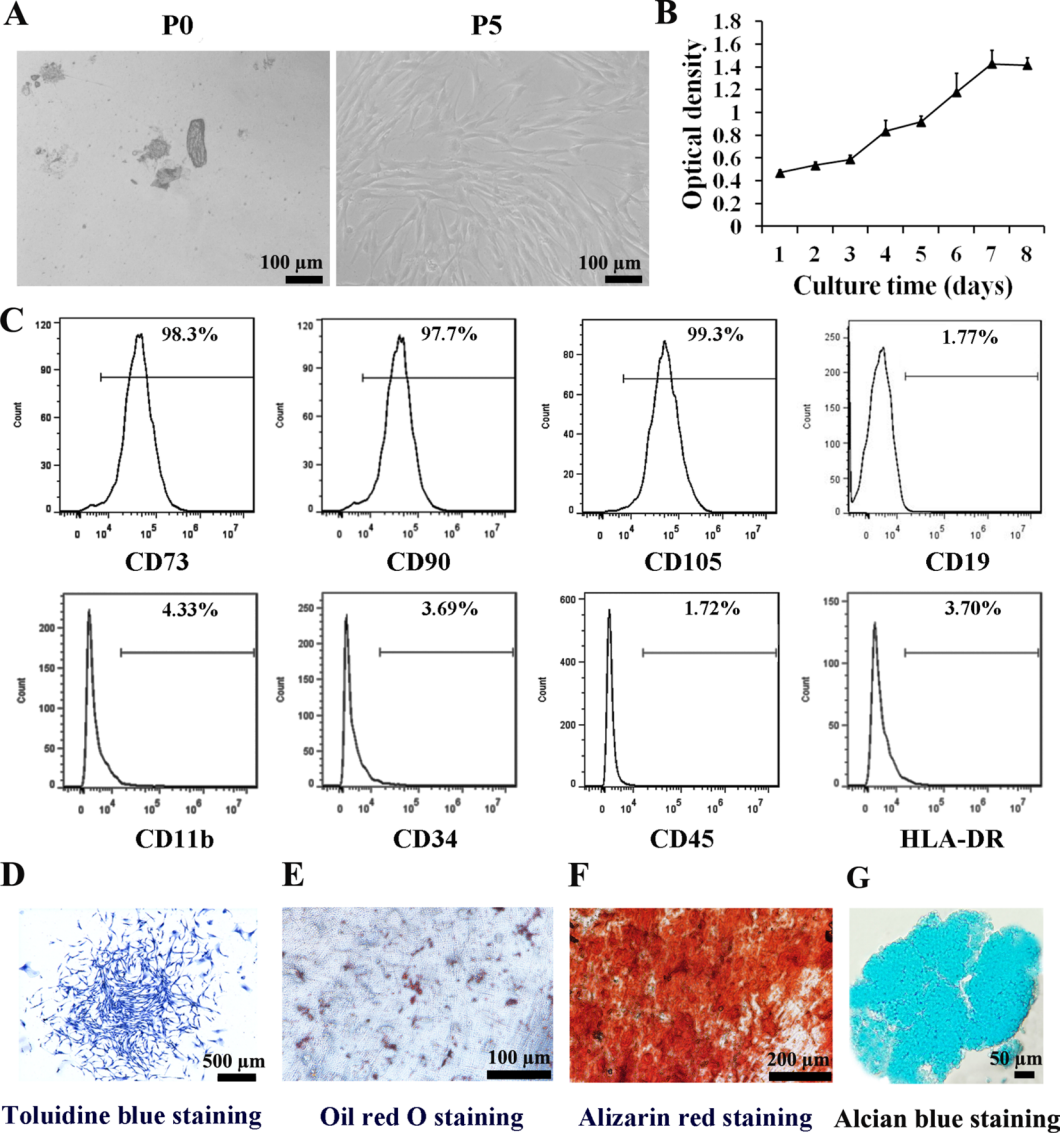
Isolation, culture, and characterization of human DPSCs. **A** P0 and P5 DPSCs were observed by an optical microscope at day 7 and day 2 (scale bars: 100 μm). **B** Growth curve of DPSCs was assessed using a CCK-8 assay. Data are expressed as mean ± SD of *n* = 8. **C** Phenotype of DPSCs was analyzed using flow cytometry. **D** CFU-F ability of DPSCs was evaluated using Toluidine blue staining at day 10 (scale bars: 500 μm). **E** Adipogenic differentiation of DPSCs was evaluated using Oil red O staining at day 21 (scale bars: 100 μm). **F** Mineralized matrix formation of DPSCs was evaluated using Alizarin red staining at day 21 (scale bars: 200 μm). G Chondrogenic differentiation of DPSCs was evaluated using Alcian blue staining at day 21 (scale bars: 50 μm)

P0 cells displayed typical fibroblastic morphology at day 7. P5 cells exhibited spindle- and triangle-shaped morphology at day 2 (Fig. 1A). Cell proliferation reached its highest at day 7, as determined by the CCK-8 kit (Fig. 1B). Flow cytometry confirmed these cells positively expressed MSC markers CD73, CD90, and CD105, while negative for hematopoietic makers CD19, CD11b, CD34, CD45, and HLA-DR (Fig. 1C). Toluidine blue staining demonstrated CFU-F ability (Fig. 1D). Under adipogenic, osteogenic, and chondrogenic conditions for 21 days, these cells formed lipid droplets (Fig. 1E), mineralized matrices (Fig. 1F) and cartilage (Fig. 1G) structures, respectively. These findings confirmed the isolated cells as MSCs.

### Insulin promotes the proliferation and osteogenic differentiation of human DPSCs

**Fig. 2:**
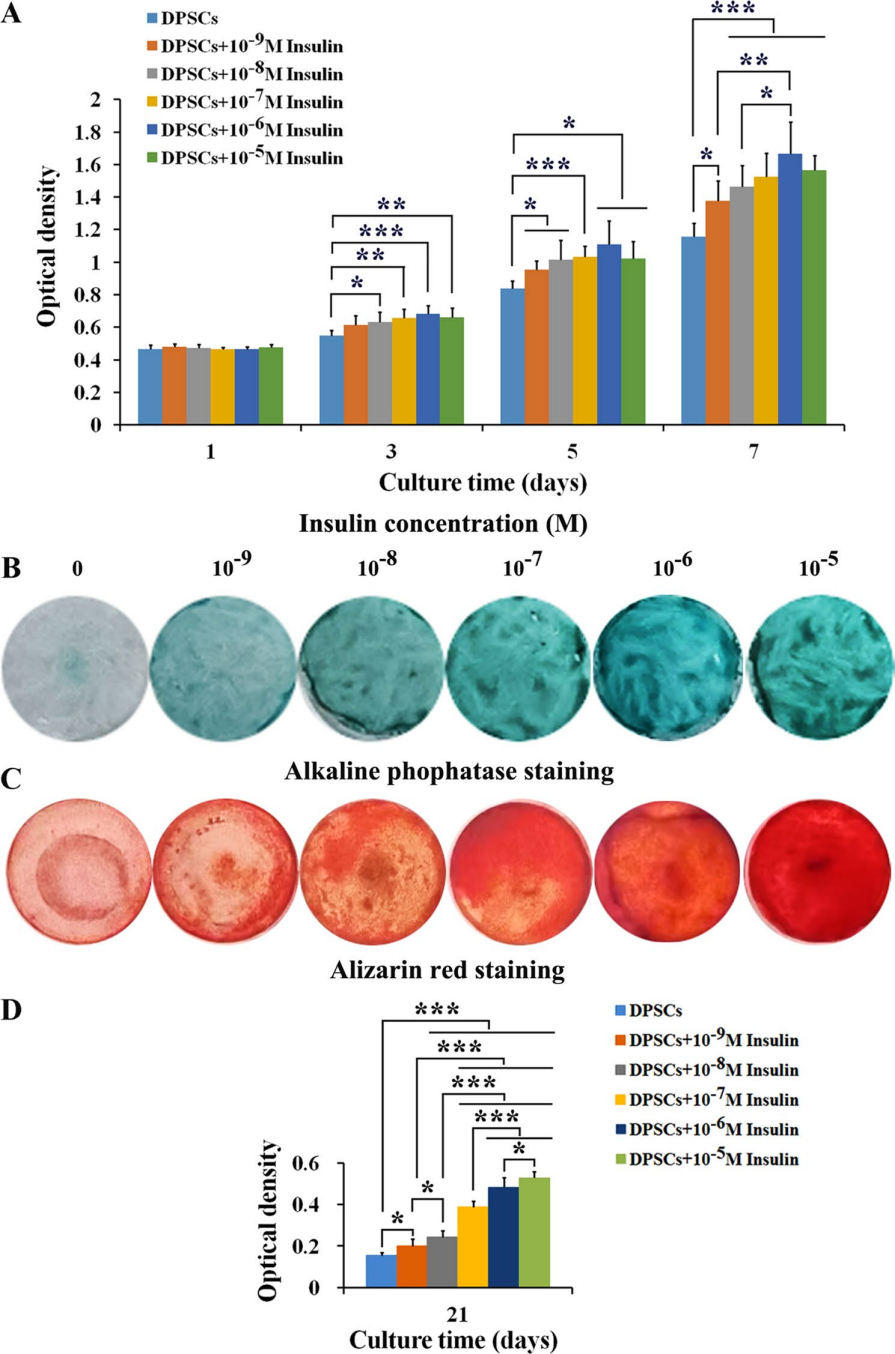
Insulin promotes the proliferation and osteogenic differentiation of human DPSCs. **A** Insulin promoted the proliferation of DPSCs in a concentration-dependent manner (*n* = 8). **B** 10^− 9^∼10^− 5^ M insulin enhanced alkaline phosphatase staining of DPSCs at day 21. **C** 10^− 9^∼10^− 5^ M insulin enhanced Alizarin red staining of DPSCs at day 21. **D** Insulin promoted the mineralized matrix formation of DPSCs in a concentration-dependent manner at day 21 (*n* = 6). Data are expressed as the mean ± SD. ^*^P < 0.05, ^**^P < 0.01, ^***^P < 0.001

Human DPSCs were treated with GM supplemented with varying concentrations of insulin (0, 10^− 9^, 10^− 8^, 10^− 7^, 10^− 6^, and 10^− 5^ M) for 1, 3, 5, and 7 days to assess the effect of insulin on cell proliferation. Notably, from day 3 onwards, except for 10^− 9^ M insulin (*P*_*3*_ > 0.05), 10^− 8^∼10^− 5^ M insulin facilitated cell proliferation (*P*_*3*_ < 0.05, *P*_*3*_ < 0.01, *P*_*3*_ < 0.001, *P*_*3*_ < 0.01). At day 5 and day 7, 10^− 9^∼10^− 5^ M insulin all significantly enhanced cell proliferation (*P*_*5*_ < 0.05, *P*_*5*_ < 0.05, *P*_*5*_ < 0.001, *P*_*5*_ < 0.05, *P*_*5*_ < 0.05) (*P*_*7*_ < 0.05, *P*_*7*_ < 0.001, *P*_*7*_ < 0.001, *P*_*7*_ < 0.001, *P*_*7*_ < 0.001). At day 7, the effect of 10^− 6^ M insulin on cell proliferation was significantly higher than those of 10^− 9^ (*P*_*7*_ < 0.01) and 10^− 8^ (*P*_*7*_ < 0.05) M insulin (Fig. 2A). These findings suggest a concentration-dependent enhancement of DPSC proliferation by insulin.

Human DPSCs were treated similarly for 21 days to evaluate the effect of insulin on the cellular osteogenic differentiation. The result showed that 10^− 9^∼10^− 5^ M insulin enhanced ALP and Alizarin red staining (Fig. 2B and C), and significantly promoted cellular mineralized matrix formation (*P*_*21*_ < 0.05, *P*_*21*_ < 0.001, *P*_*21*_ < 0.0001, *P*_*21*_ < 0.001, *P*_*21*_ < 0.001). There were significant differences in the mineralized matrix formation between different insulin concentrations, and higher concentration insulin showed more mineralized matrix formation (Fig. 2D). These results demonstrate that insulin not only boosts DPSC proliferation but also enhances their osteogenic differentiation in a concentration-dependent manner, 10^− 6^ M insulin was selected for subsequent experiments.

### 10^− 6^ M insulin promotes the osteogenic differentiation of human DPSCs

**Fig. 3:**
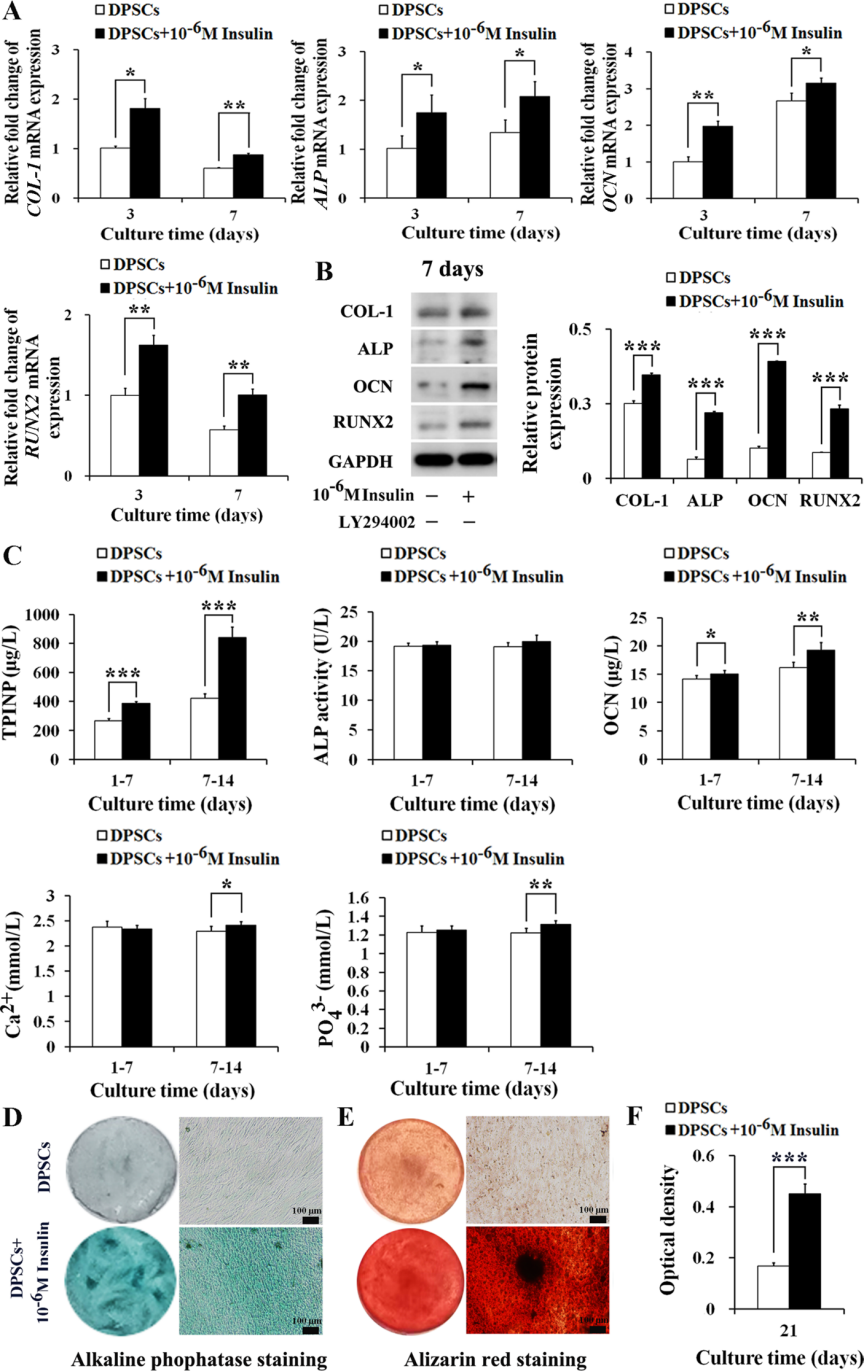
10^− 6^ M insulin promotes the osteogenic differentiation of human DPSCs. **A** 10^− 6^ M insulin up-regulated the mRNA levels of *COL-1*, *ALP*, *OCN*, and *RUNX2* in DPSCs at day 3 and day 7 (*n* = 3). **B** 10^− 6^ M insulin promoted the protein expressions of COL-1, ALP, OCN, and RUNX2 in DPSCs at day 7 (*n* = 3). Representative western blotting (left) and quantification analysis (right). Full-length blots/gels are presented in Supplementary Fig. 1. **C** 10^− 6^ M insulin increased the secretion of extracellular bone metabolism and biochemical markers in DPSCs at day 1–7 and day 7–14 (*n* = 6). **D** 10^− 6^ M insulin enhanced alkaline phosphatase staining of DPSCs at day 21 (scale bars: 100 μm). **E** 10^− 6^ M insulin enhanced Alizarin red staining of DPSCs at day 21 (scale bars: 100 μm). **F** 10^− 6^ M insulin promoted the mineralized matrix formation of DPSCs at day 21 (*n* = 6). Data are expressed as the mean ± SD. ^*^P < 0.05, ^**^P < 0.01, ^***^P < 0.001

Given insulin promoting the proliferation and osteogenic differentiation of human DPSCs, we further investigated the effect of 10^− 6^ M insulin on the osteogenic differentiation of DPSCs. The results showed that 10^− 6^ M insulin treatment markedly increased the mRNA levels of *COL-1* (*P*_*3*_ < 0.05, *P*_*7*_ < 0.01), *ALP* (*P*_*3*_ < 0.05, *P*_*7*_ < 0.05), *OCN* (*P*_*3*_ < 0.01, *P*_*7*_ < 0.01) and *RUNX2* (*P*_*3*_ < 0.01, *P*_*7*_ < 0.01) in DPSCs at day 3 and day 7 (Fig. 3A). Correspondingly, protein expressions of COL-1 (*P*_*7*_ < 0.001), ALP (*P*_*7*_ < 0.001), OCN (*P*_*7*_ < 0.001), and RUNX2 (*P*_*7*_ < 0.001) were significantly enhanced by 10^− 6^ M insulin treatment at day 7 (Fig. 3B).

We also evaluated the impact of insulin on extracellular bone metabolism and biochemical markers, along with the cells’ mineralized matrix formation capability. From day 1 to 7 and day 7 to 14, 10^− 6^ M insulin notably boosted the secretion of extracellular TPINP (*P*_*1 − 7*_<0.001, *P*_*7 − 14*_<0.001) and OCN (*P*_*1 − 7*_<0.05, *P*_*7 − 14*_<0.01), with ALP secretion remaining unchanged (*P*_*1 − 7*_>0.05, *P*_*7 − 14*_>0.05). Additionally, significant increases were observed in the extracellular concentrations of Ca^2+^ (*P*_*7 − 14*_<0.05) and PO_4_^3−^ (*P*_*7 − 14*_<0.01) at day 7–14 under 10^− 6^ M insulin treatment (Fig. 3C).

Furthermore, enhancements in osteogenic differentiation were corroborated by intensified ALP activity and Alizarin red staining at day 21 (Fig. 3D and E). Quantitative analysis of the Alizarin red staining confirmed that 10^− 6^ M insulin substantially promoted mineralized matrix formation at day 21 (*P*_*21*_ < 0.001) (Fig. 3F). Collectively, these findings affirm that 10^− 6^ M insulin substantially augments the osteogenic differentiation in human DPSCs, showcasing its potential for enhancing bone regeneration therapies.

### 10^− 6^ M insulin inhibits the gene and protein expressions of IIS-related receptors and substrates in human DPSCs

**Fig. 4:**
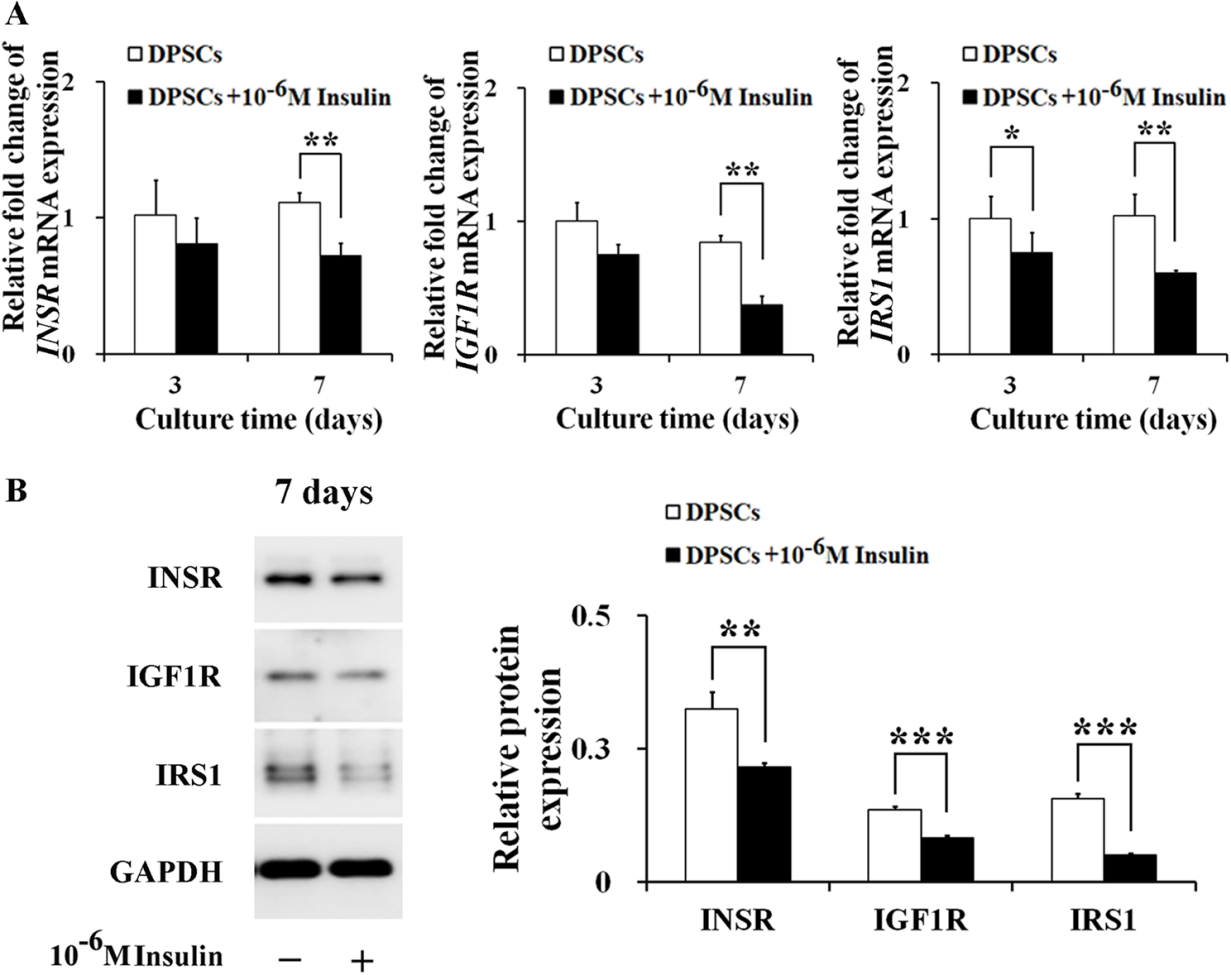
10^− 6^ M insulin inhibits the gene and protein expressions of the IIS-related receptors and substrates in human DPSCs. **A** 10^− 6^ M insulin down-regulated the mRNA levels of *INSR*, *IGF1R*, and *IRS1* in DPSCs at day 3 and day 7. **B** 10^− 6^ M insulin inhibited the protein expressions of INSR, IGF1R, and IRS1 in DPSCs at day 7. Representative western blotting (left) and quantification analysis (right). Full-length blots/gels are presented in Supplementary Fig. 2. Data are expressed as the mean ± SD of *n* = 3. ^*^P < 0.05, ^**^P < 0.01, ^***^P < 0.001

To further understand how 10^− 6^ M insulin enhances the proliferation and osteogenic differentiation in human DPSCs, we focused on the gene and protein expression levels of key components in IIS pathway, such as INSR, IGF1R, and IRS1. Interestingly, 10^− 6^ M insulin treatment resulted in significant reduction in the mRNA level of *IRS1* at both day 3 and day 7 (*P*_*3*_ < 0.05, *P*_*7*_ < 0.01), suggesting an inhibitory effect early in the signaling pathway. Additionally, the mRNA levels of *INSR* (*P*_*7*_ < 0.01) and *IGF1R* (*P*_*7*_ < 0.01) were also obviously reduced by 10^− 6^ M insulin at day 7 (Fig. 4A). Protein analysis corroborated these findings, 10^− 6^ M insulin treatment markedly reduced the protein levels of INSR (*P*_*7*_ < 0.01), IGF1R (*P*_*7*_ < 0.001), and IRS1 (*P*_*7*_ < 0.001) at day 7 (Fig. 4B). These results imply that 10^− 6^ M insulin may modulate the proliferation and osteogenic differentiation of DPSCs through a mechanism involving the suppression of IIS.

### LY294002 attenuates the responsiveness of 10^− 6^ M insulin to the IIS/PI3K/AKT/mTOR pathway axis in human DPSCs

**Fig. 5:**
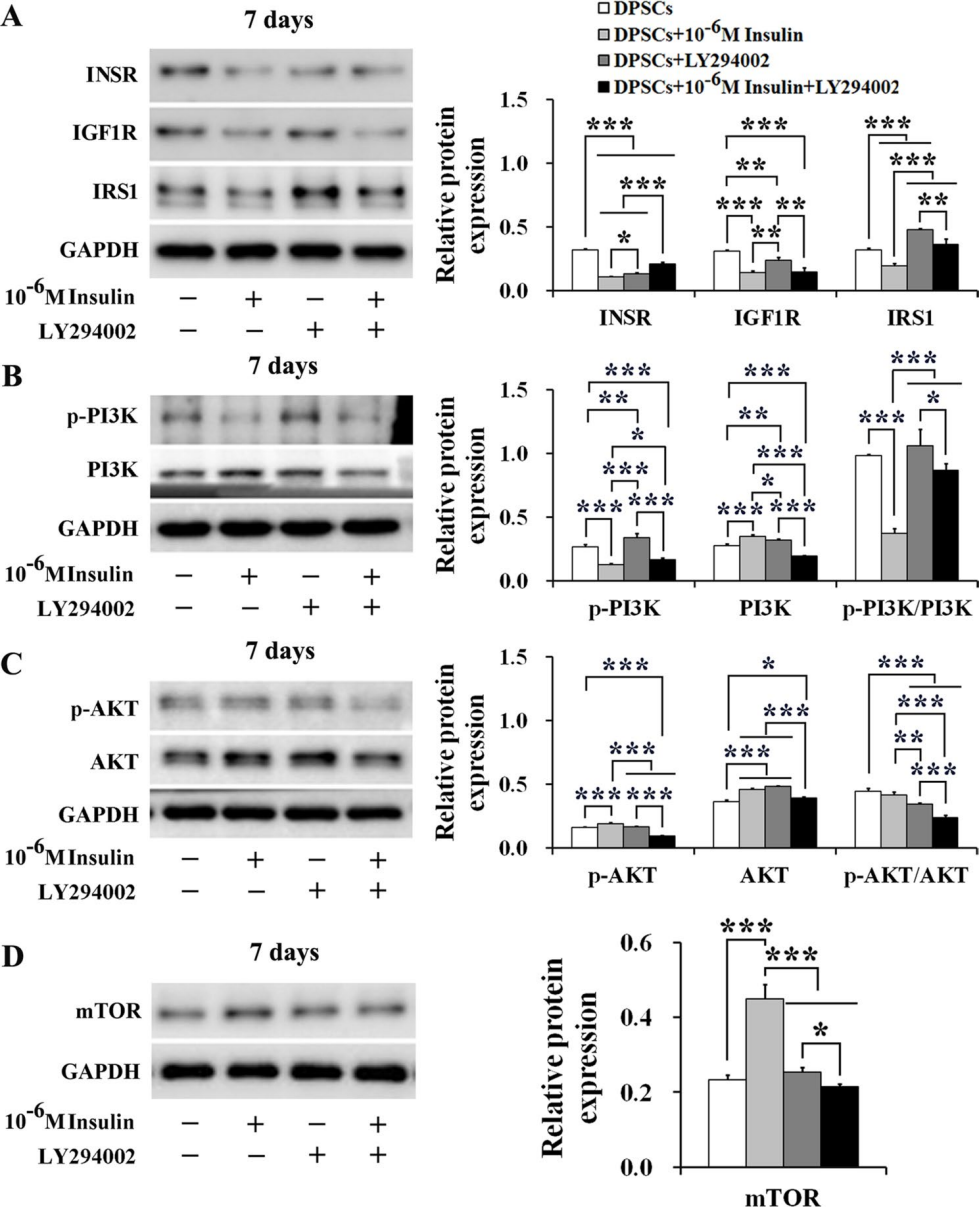
LY294002 attenuates the responsiveness of 10^− 6^ M insulin to the IIS/PI3K/AKT/mTOR pathway axis in human DPSCs. **A** LY294002 attenuated the inhibiting effect of 10^− 6^ M insulin on the protein expressions of INSR, IGF1R, and IRS1 in DPSCs at day 7. **B** and **C** LY294002 attenuated the responsiveness of 10^− 6^ M insulin to the PI3K/AKT pathway in DPSCs at day 7. **D** LY294002 attenuated the promoting effect of 10^− 6^ M insulin on the protein expression of mTOR in DPSCs at day 7. Representative western blotting (left) and quantification analysis (right). Data are expressed as the mean ± SD of *n* = 3. Full-length blots/gels are presented in Supplementary Figs. 3–6. ^*^P < 0.05, ^**^P < 0.01, ^***^P < 0.001

In physiological state, insulin secreted by the body enhances IIS signal transduction through PI3K/AKT pathway and activation of mTOR, maintaining normal cell growth, proliferation, and differentiation [32]. However, our RT-qPCR and Western blotting data revealed that 10^− 6^ M insulin significantly suppressed IIS in human DPSCs. Given that LY294002 is a known inhibitor of the PI3K/AKT pathway, we assessed the interaction between 10^− 6^ M insulin and 10 µM LY294002 on the IIS/PI3K/AKT/mTOR axis in human DPSCs at day 7. Our results showed that 10^− 6^ M insulin significantly reduced the protein levels of INSR (*P*_*7*_ < 0.001), IGF1R (*P*_*7*_ < 0.001), and IRS1 (*P*_*7*_ < 0.001) in human DPSCs. In contrast, the combination of 10^− 6^ M insulin and LY294002 notably increased the protein levels of INSR (*P*_*7*_ < 0.05), IGF1R (*P*_*7*_ < 0.01), and IRS1 (*P*_*7*_ < 0.001) compared to 10^− 6^ M insulin alone. Additionally, LY294002 alone significantly decreased the protein levels of INSR (*P*_*7*_ < 0.001) and IGF1R (*P*_*7*_ < 0.01), but increased IRS1 level (*P*_*7*_ < 0.001) compared to the control group (Fig. 5A).

Further analysis showed that 10^− 6^ M insulin significantly diminished phosphorylated PI3K level (*P*_*7*_ < 0.001), obviously enhanced total PI3K levels (*P*_*7*_ < 0.001), and markedly reduced the ratio of phosphorylated PI3K/total PI3K (*P*_*7*_ < 0.001). However, LY294002 reversed these effects of insulin, obviously enhanced phosphorylated PI3K levels (*P*_*7*_ < 0.05) and decreased total PI3K levels (*P*_*7*_ < 0.001), thereby increasing the ratio of phosphorylated PI3K/total PI3K (*P*_*7*_ < 0.001). Additionally, LY294002 treatment alone significantly promoted phosphorylated PI3K level (*P*_*7*_ < 0.01) and total PI3K level (*P*_*7*_ < 0.01) compared to the control group. There was no effect on the ratio of the phosphorylated PI3K/total PI3K (*P*_*7*_ > 0.05) (Fig. 5B).

Unlike PI3K, AKT responses to 10^− 6^ M insulin and LY294002 were different, while 10^− 6^ M insulin alone markedly promoted the phosphorylation AKT level (*P*_*7*_ < 0.001) and total AKT level (*P*_*7*_ < 0.001). Although the ratio of the phosphorylated AKT/total AKT was reduced, the difference was not significant (*P*_*7*_ > 0.05). However, LY294002 reversed these effects of insulin, markedly inhibited the phosphorylated AKT level (*P*_*7*_ < 0.001) and total AKT level (*P*_*7*_ < 0.001), and obviously reduced the ratio of the phosphorylated AKT/total AKT (*P*_*7*_ < 0.001). Furthermore, LY294002 alone significantly promoted total AKT level (*P*_*7*_ < 0.001) and significantly reduced the ratio of the phosphorylated AKT/total AKT compared to the control group (*P*_*7*_ < 0.001) (Fig. 5C).

Finally, we assessed the effect of 10^− 6^ M insulin on mTOR activity in human DPSCs, a downstream regulator of PI3K/AKT pathway. Results showed that 10^− 6^ M insulin alone significantly increased mTOR protein levels in DPSCs at day 7 (*P*_*7*_ < 0.001), whereas the addition of LY294002 obviously reduced mTOR protein levels induced by 10^− 6^ M insulin (*P*_*7*_ < 0.001) (Fig. 5D). These findings suggest that LY294002 can attenuate the effects of 10^− 6^ M insulin on the IIS/PI3K/AKT/mTOR pathway in human DPSCs, potentially influencing insulin-mediated responses in osteogenic differentiation.

### LY294002 attenuates the promoting effect of 10^− 6^ M insulin on the proliferation and osteogenic differentiation of human DPSCs

**Fig. 6:**
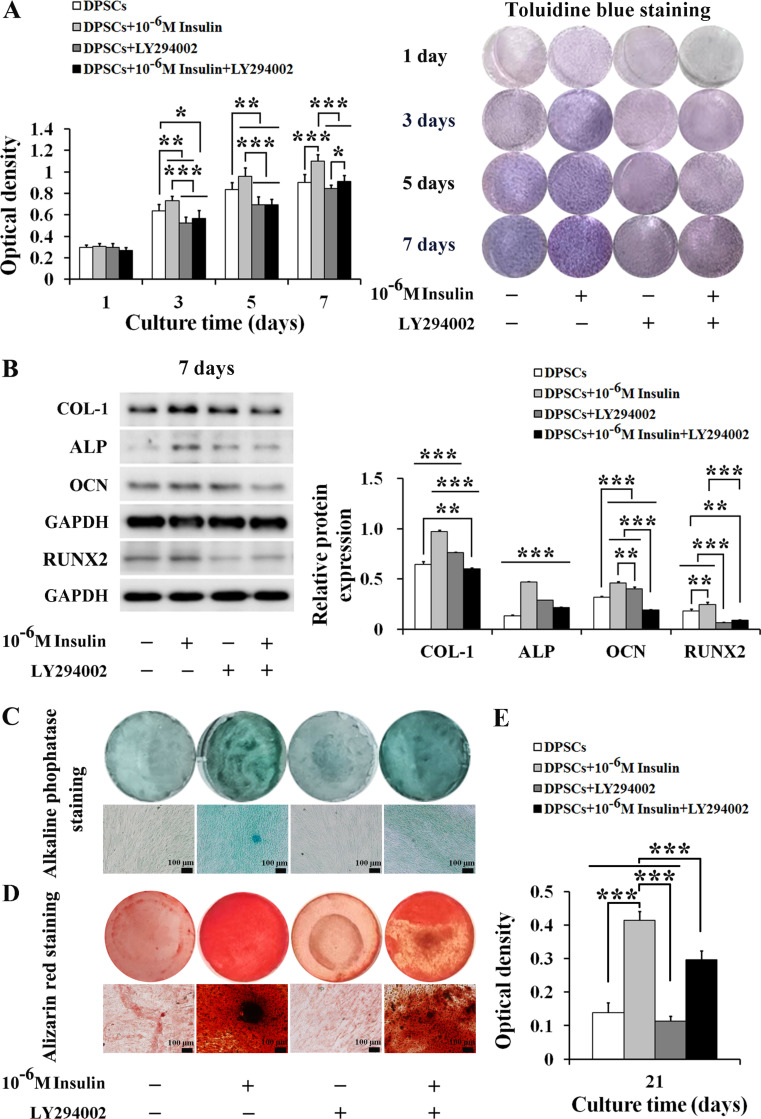
LY294002 attenuates the promoting effect of 10^− 6^ M insulin on the proliferation and osteogenic differentiation of human DPSCs. **A** LY294002 attenuated the promoting effect of 10^− 6^ M insulin on the proliferation of DPSCs at day 1, 3, 5, and 7. (*n* = 8). **B** LY294002 attenuated the promoting effect of 10^− 6^ M insulin on the protein expression of COL-1, ALP, OCN and RUNX2 in DPSCs at day 7 (*n* = 3). Representative western blotting (left) and quantification analysis (right). Full-length blots/gels are presented in Supplementary Fig. 7. **C** LY294002 attenuated the enhancing effect of 10^− 6^ M insulin on alkaline phosphatase staining of DPSCs at day 21 (scale bars: 100 μm). **D** LY294002 attenuated the enhancing effect of 10^− 6^ M insulin on Alizarin red staining of DPSCs at day 21 (scale bars: 100 μm). **E** LY294002 attenuated the promoting effect of 10^− 6^ M insulin on the mineralized matrix formation of DPSCs at day 21. Data are expressed as the mean ± SD of *n* = 6. ^*^P < 0.05, ^**^P < 0.01, ^***^P < 0.001

Our data have established that 10^− 6^ M insulin facilitates the proliferation and osteogenic differentiation of human DPSCs. This study further explored the impacts of the combined 10^− 6^ M insulin with 10 µM LY294002 on these cellular processes through the IIS/PI3K/AKT/mTOR pathway. The results found that at day 3 (*P*_*3*_ < 0.01), day 5 (*P*_*5*_ < 0.01), and day 7 (*P*_*7*_ < 0.001), 10^− 6^ M insulin significantly promoted DPSCs’ proliferation. However, the combination of insulin with LY294002 notably inhibited this proliferation at all time points [day 3 (*P*_*3*_ < 0.001), day 5 (*P*_*5*_ < 0.001) and day 7 (*P*_*7*_ < 0.001)]. LY294002 alone also significantly reduced cell proliferation compared to the control group at day 3 (*P*_*3*_ < 0.01) and day 5 (*P*_*5*_ < 0.01), corroborating the inhibitory effect observed with Toluidine blue staining (Fig. 6A).

Consistent with the proliferation patterns, 10^− 6^ M insulin alone significantly increased the protein levels of osteogenic markers COL-1 (*P*_*7*_ < 0.001), ALP (*P*_*7*_ < 0.001), OCN (*P*_*7*_ < 0.001), and RUNX2 (*P*_*7*_ < 0.01) in DPSCs at day 7. However, the co-treatment with LY294002 significantly suppressed the expression of COL-1 (*P*_*7*_ < 0.001), ALP (*P*_*7*_ < 0.001), OCN (*P*_*7*_ < 0.001), and RUNX2 (*P*_*7*_ < 0.001) induced by 10^− 6^ M insulin. Notably, LY294002 alone enhanced the levels of COL-1 (*P*_*7*_ < 0.001), ALP (*P*_*7*_ < 0.001), and OCN (*P*_*7*_ < 0.001), but reduced RUNX2 (*P*_*7*_ < 0.001) compared to the control group (Fig. 6B).

At day 21, 10^− 6^ M insulin obviously enhanced ALP staining, Alizarin red staining, and mineralized matrix formation (*P*_*21*_ < 0.001). However, LY294002 significantly attenuated these staining and mineralized matrix formation induced by 10^− 6^ M insulin (*P*_*21*_ < 0.001) (Fig. 6C, D, and E). These findings suggest that LY294002 can effectively modulate the enhancing effects of 10^− 6^ M insulin on the proliferation and osteogenic differentiation of human DPSCs by interfering with the IIS/PI3K/AKT/mTOR pathway.

### 10^− 6^ M insulin promotes the bone formation capability of human DPSCs

**Fig. 7:**
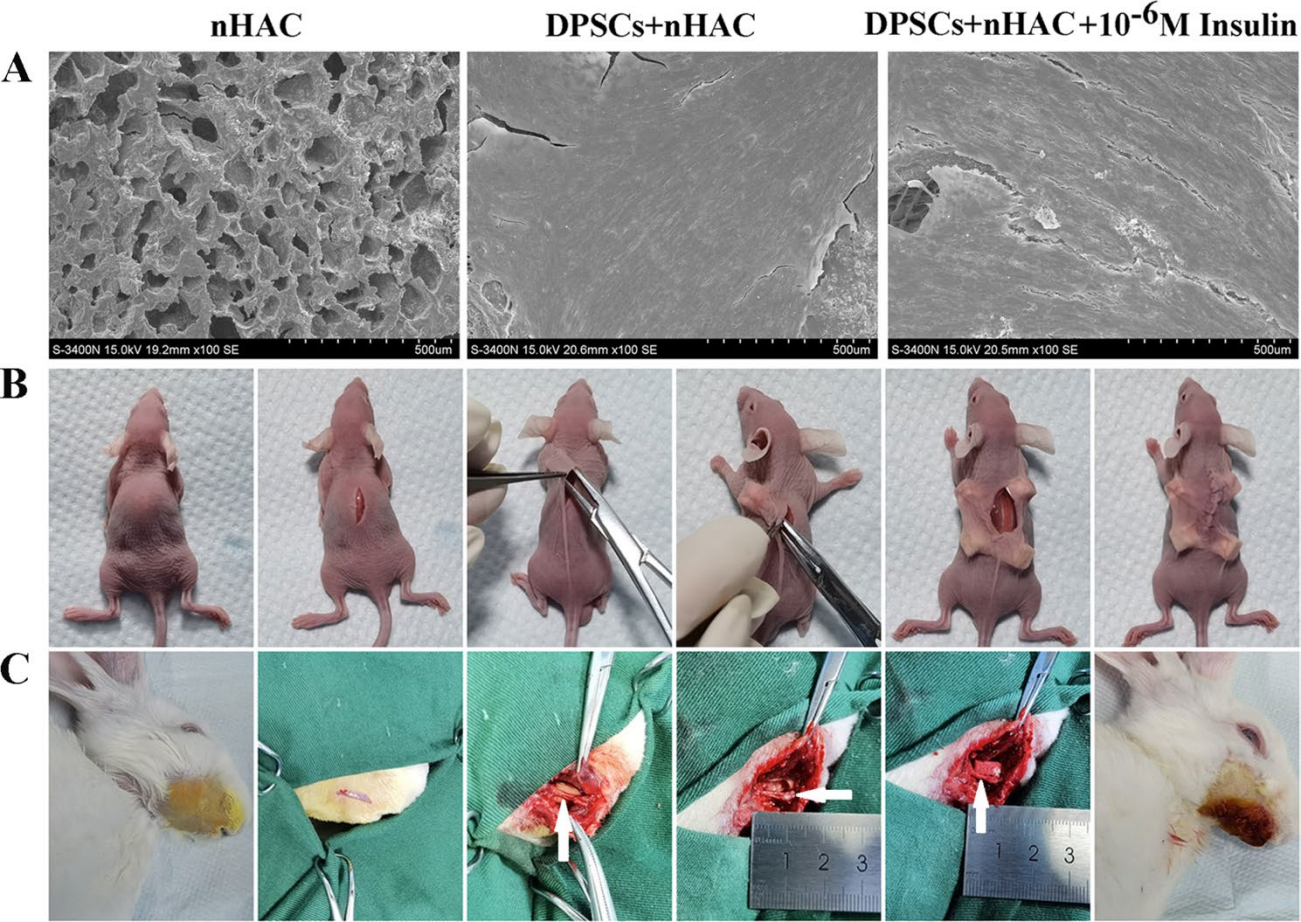
Scanning electron microscope observation and the schematic diagram of the surgery. **A** Scanning electron microscope observation of the constructs at day 7 (scale bars: 500 μm). **B** The constructs were subcutaneously implanted into the backs of SCID mice. **C** The constructs were implanted into the jawbone defects in New Zealand white rabbits. The white arrow indicates the mental nerve

**Fig. 8:**
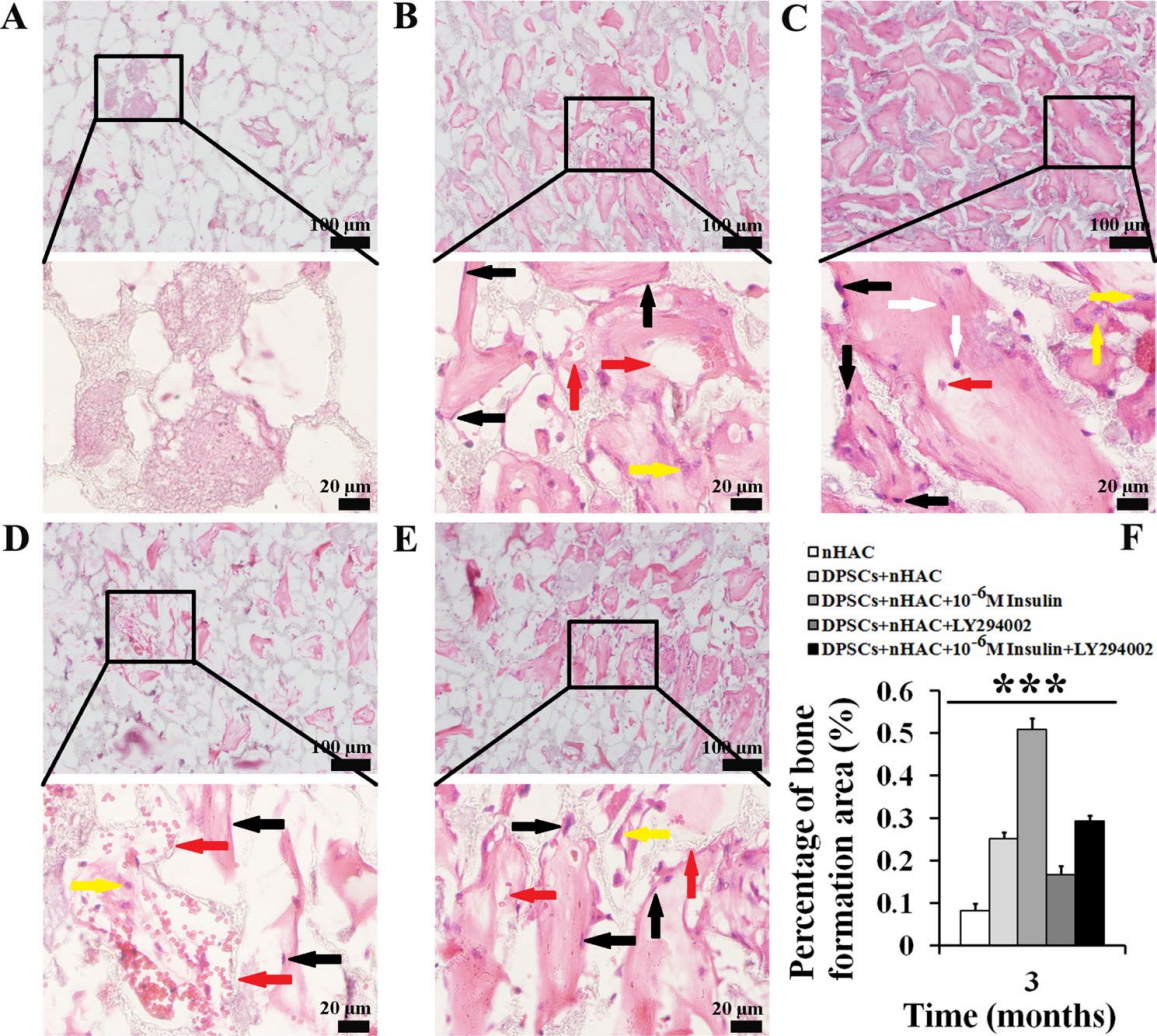
Hematoxylin and eosin staining after the constructs were implanted subcutaneously into the backs of SCID mice for 3 months. **A** nHAC group. **B** DPSCs + nHAC group. **C** DPSCs + nHAC + 10^− 6^ M Insulin group. **D** DPSCs + nHAC + LY294002 group. **E** DPSCs + nHAC + 10^− 6^ M Insulin + LY294002 group. The image below (scale bars: 20 μm) is respectively a magnification of the image above in Figure A-E (scale bars: 100 μm). **F** Percentage of bone formation area. The black arrow indicates osteoblasts, the white arrow indicates osteocytes in bone lacuna, the yellow arrow indicates osteoclasts, the red arrow indicates blood vessels. Data are expressed as the mean ± SD of *n* = 6. ^***^P < 0.001

**Fig. 9:**
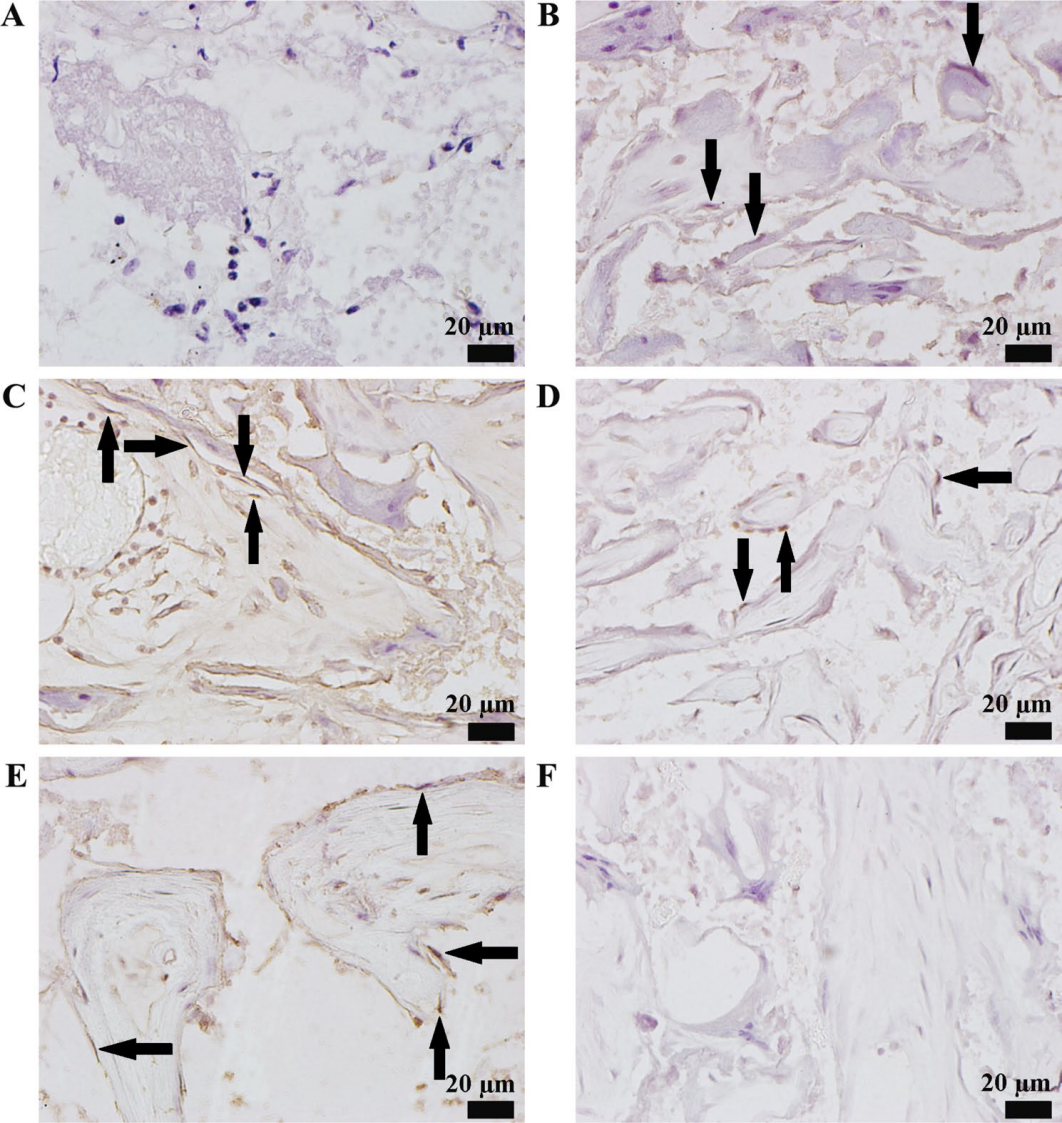
Immunohistochemical staining of OCN after the constructs were implanted subcutaneously into the backs of SCID mice for 3 months. **A** nHAC group. **B** DPSCs + nHAC group. **C** DPSCs + nHAC + 10^− 6^ M Insulin group. **D** DPSCs + nHAC + LY294002 group. **E** DPSCs + nHAC + 10^− 6^ M Insulin + LY294002 group. **F** PBS replaced the primary antibody. Scale bars: 20 μm. The black arrow indicates osteoblasts

To explore the effect of 10^− 6^ M insulin on the bone formation capability of human DPSCs in vivo, we seeded DPSCs into nHAC scaffolds. These constructs were cultured in GM with and without 10^− 6^ M insulin for 7 days. Scanning electron microscopy confirmed robust cell adhesion, proliferation, and differentiation on the scaffolds (Fig. 7A), consistent with prior findings [34]. Next, the constructs were implanted subcutaneously into the backs of SCID mice (Fig. 7B) and into jawbone defects in New Zealand white rabbits (Fig. 7C). Three months post-implantation, we evaluated bone formation. In SCID mice, H&E staining revealed new bone formation in all experimental groups (Fig. 8A–E). Moreover, the percentage of bone formation areas in the DPSCs + nHAC group (*P* < 0.001), DPSCs + nHAC + 10^− 6^ M Insulin group (*P* < 0.001), DPSCs + nHAC + LY294002 group (*P* < 0.001), and DPSCs + nHAC + 10^− 6^ M Insulin + LY294002 group (*P* < 0.001) were significantly higher than that in the nHAC group. This value in the DPSCs + nHAC + 10^− 6^ M Insulin group was significantly higher than those in the DPSCs + nHAC group (*P* < 0.001), DPSCs + nHAC + LY294002 group (*P* < 0.001) and DPSCs + nHAC + 10^− 6^ M Insulin + LY294002 group (*P* < 0.001) (Fig. 8F). Immunohistochemical analysis indicated that the nHAC group exhibited negligible OCN expression despite some bone formation (Fig. 9A). In contrast, OCN was positively expressed in the DPSCs + nHAC group (Fig. 9B), DPSCs + nHAC + 10^− 6^ M Insulin group (Fig. 9C), DPSCs + nHAC + LY294002 group (Fig. 9D), and DPSCs + nHAC + 10^− 6^ M Insulin + LY294002 group (Fig. 9E). Controls using PBS in place of the primary antibody confirmed the specificity of staining (Fig. 9F).

**Fig. 10:**
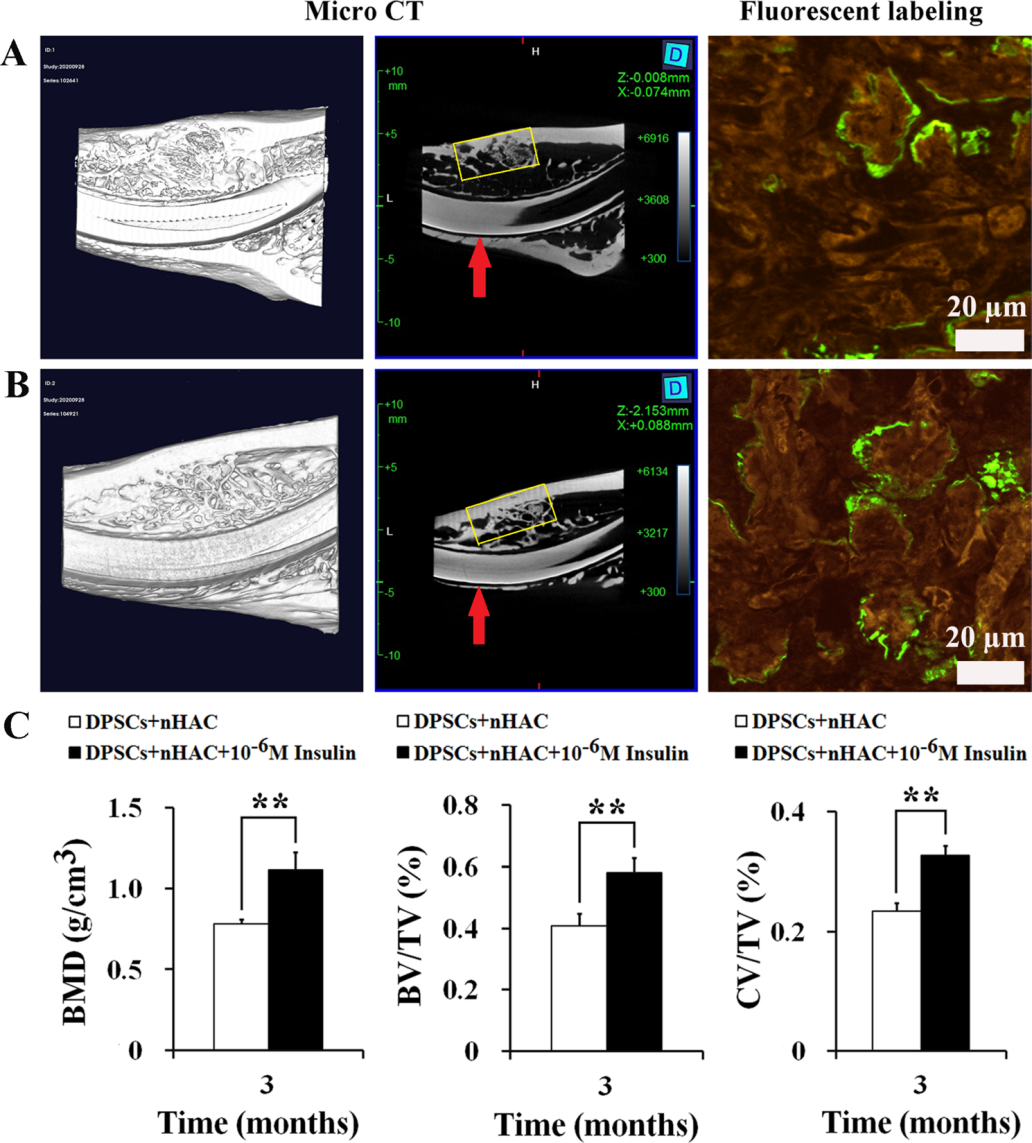
Micro-CT and confocal laser scanning microscopy observation after the constructs were implanted into the jawbone defects in rabbits for 3 months. **A** DPSCs + nHAC group. **B** DPSCs + nHAC + 10^− 6^ M Insulin group. Calcein fluorescence was displayed in green under confocal laser scanning microscopy (scale bars: 20 μm). **C** Bone mineral density (BMD), bone volume/total volume (BV/TV), and cortical bone volume/total volume (CV/TV) were evaluated by micro-CT measurements. Data are expressed as the mean ± SD of *n* = 3. The red arrow indicates the incisors of the mandible. The yellow box indicates the jawbone defects. ^**^P < 0.01

**Fig. 11:**
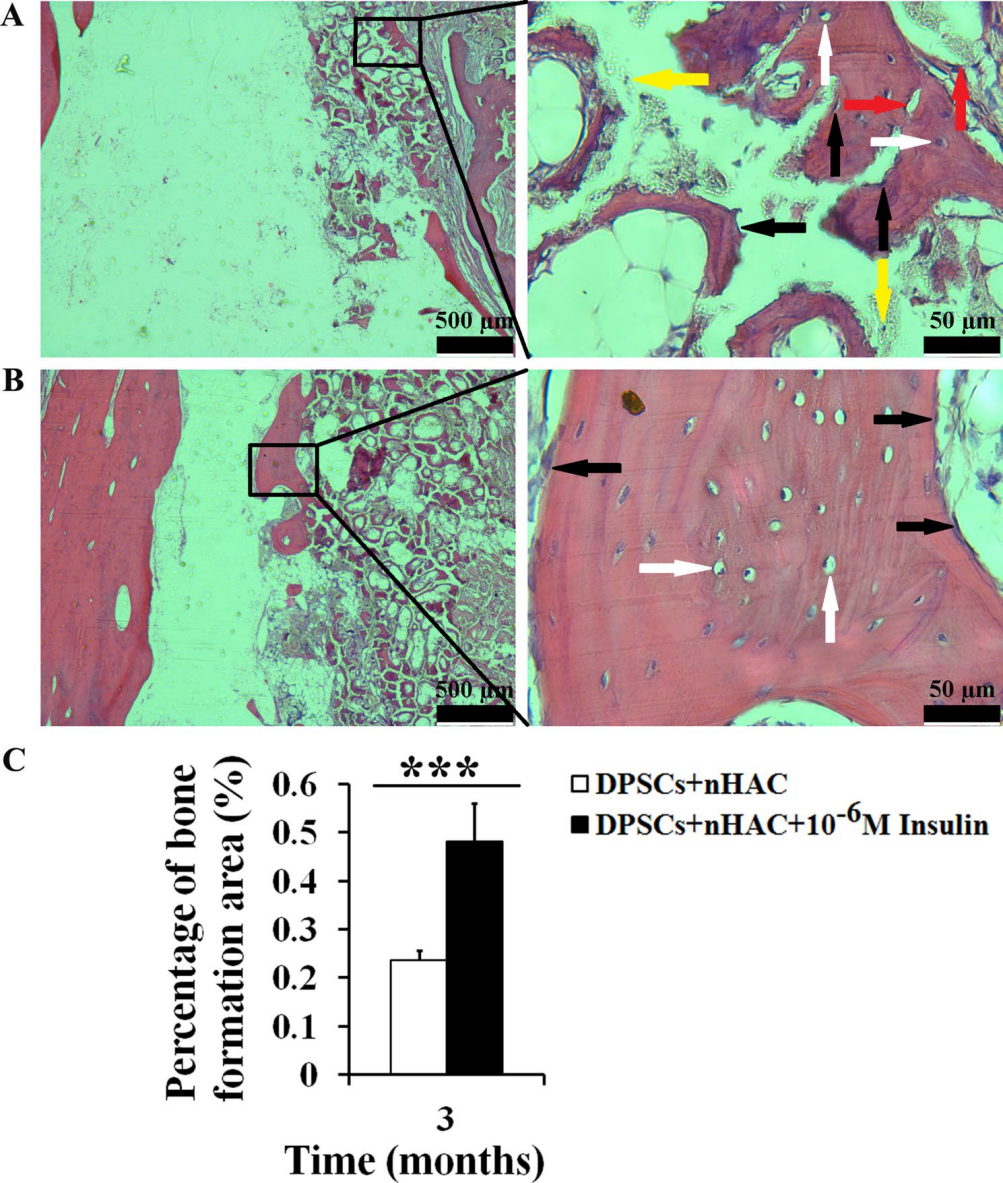
Hematoxylin and eosin staining after the constructs were implanted into the jawbone defects in rabbits for 3 months. **A** DPSCs + nHAC group. **B** DPSCs + nHAC + 10^− 6^ M Insulin group. The image on the right (scale bars: 50 μm) is a magnification of the image on the left (scale bars: 500 μm). **C** Percentage of bone formation area. Data are expressed as the mean ± SD of *n* = 6. The black arrow indicates osteoblasts, the white arrow indicates osteocytes in bone lacuna, the yellow arrow indicates osteoclasts, the red arrow indicates blood vessels. ^***^P < 0.001

**Fig. 12:**
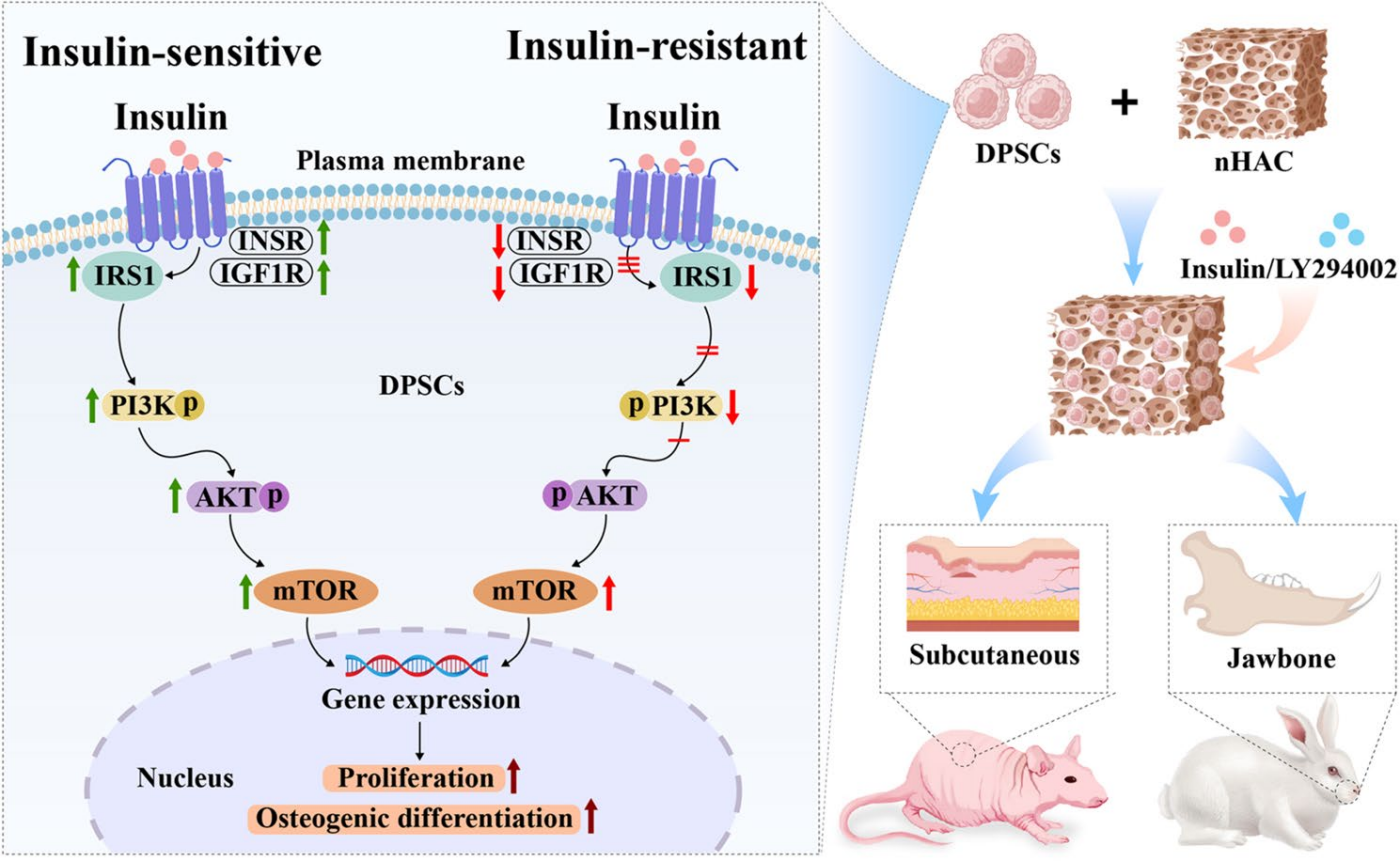
Schematic diagram of the mechanism by which 10^− 6^ M insulin impacts the bone formation capability of human DPSCs

In the rabbit jawbone defects, micro-CT reconstructions demonstrated mineralized tissue coverage over the defects in both DPSCs + nHAC and DPSCs + nHAC + 10^− 6^ M Insulin groups, the more organized bone formations were observed in the latter (Fig. 10A and B). To quantify the bone formation, the bone mineral density (BMD), bone volume/total volume (BV/TV), and cortical bone volume/total volume (CV/TV) were measured. 10^− 6^ M Insulin obviously enhanced BMD (*P* < 0.01), BV/TV (*P* < 0.01), and CV/TV (*P* < 0.01) in DPSCs + nHAC constructs (Fig. 10C). Confocal laser scanning microscopy revealed calcein fluorescence, indicating active bone formation in both groups (Fig. 10A and B). H&E staining confirmed the presence of new bone, characterized by abundant vascularization, active osteoblasts lining the bone surfaces, randomly arranged osteocytes in the lacunae, and osteoclasts involved in scaffold degradation (Fig. 11A-B). Statistical analysis showed a significantly higher percentage of bone formation area in the 10^− 6^ M insulin-treated group compared to the DPSCs + nHAC group (*P* < 0.001) (Fig. 11C). Furthermore, the schematic diagram shown in Fig. 12 outlines the mechanism by which 10^− 6^ M insulin impacts the bone formation capability of human DPSCs.

## Discussion

This study underscores that insulin plays essential roles in human DPSCs’ proliferation, osteogenic differentiation, and bone formation practices. Specifically, 10^− 6^ M insulin induces insulin-resistance in human DPSCs, resulting in impairments in proximal insulin signaling events while maintaining the activity of distal pathway components, and continues to enhance the proliferation, osteogenic differentiation, and bone formation capability of human DPSCs via gradually attenuating the IIS/PI3K/AKT/mTOR pathway. This is a novel finding, as the specific effects of insulin on human DPSCs have not been previously explored in detail in vitro and in vivo.

Insulin, with a physiological concentration ranging from 10^− 9^ to 10^− 7^ M, is an anabolic agent in bone [17, 46] and has commonly been found to promote the proliferation and osteogenic differentiation of various cells types [20, 26–28, 34, 47, 48]. For instance, Yang [26] showed that the physiological-concentration insulin promoted the proliferation of MG-63 cells in a concentration (10^− 9^, 10^− 8^, and 10^− 7^ M)- and time (24, 48, and 72 h)-dependent manner, 10^− 8^ M insulin showed the most significant proliferative effect after 72 h and significantly enhanced the osteogenic differentiation of MG-63 cells. Additionally, Kream [47] reported that the physiological-concentration insulin (10^− 9^, 10^− 8^, and 10^− 7^ M) appeared to increase bone collagen synthesis by a direct effect on the osteoblast, whereas the high-concentration insulin (10^− 6^ M) had an additional action to increase the replication of collagen-synthesizing cells. Li [28] demonstrated that insulin enhanced the proliferation of rat spinal ligament cells in a time (24, 48, 72, and 96 h)- and concentration (10^− 10^, 10^− 9^, 10^− 8^, 10^− 7^, and 10^− 6^ M)-dependent manner. The high-concentration insulin (10^− 6^ M) not only showed the maximum proliferative effect after 4 days but also potentiated BMP-2-induced osteogenic differentiation. Our data align with these observations, showing that insulin enhanced the proliferation of human DPSCs in a concentration-dependent manner. Specifically, 10^− 9^ M insulin significantly enhanced DPSC proliferation from day 5, whereas 10^− 8^∼10^− 5^ M insulin began to have a significant proliferative effect from day 3. Notably, at day 7, the proliferation of cells treated with 10^− 6^ M insulin was significantly higher than those of cells treated with 10^− 9^ and 10^− 8^ M insulin, indicating the different concentrations insulin gradually showed significant difference in promoting the proliferation of human DPSCs over time. Although the difference was not significant, the proliferation of cells treated with 10^− 7^ and 10^− 5^ M insulin was lower than that of cells treated with 10^− 6^ M insulin, suggesting the most obvious proliferative effect was observed in 10^− 6^ M insulin within 7 days.

Moreover, our results also demonstrated that insulin obviously enhanced ALP and Alizarin red staining of human DPSCs and significantly promoted mineralized matrix formation of human DPSCs in a concentration-dependent manner by day 21, while the high-concentration insulin (10^− 6^ and 10^− 5^ M) particularly promoted mineralized matrix formation in human DPSCs. These findings suggest that insulin has a direct anabolic effect on human DPSCs, promoting both cell proliferation and osteogenic differentiation. High-concentration insulin appears to be more effective in promoting cell proliferation and osteogenic differentiation in vitro. In conclusion, our study contributes to the understanding of insulin’s role in bone biology and highlights its potential as a therapeutic agent for promoting the proliferation and osteogenic differentiation of human DPSCs.

Based on the observation that high-concentration insulin had a more pronounced impact on the proliferation and osteogenic differentiation of human DPSCs, we chose to focus on 10^− 6^ M insulin for our insulin treatment in subsequent experiments. Our results found that 10^− 6^ M insulin significantly increased the mRNA (3 and 7 days) and protein (7 days) expressions of markers associated with osteogenic differentiation, obviously enhanced the secretion of bone metabolism and biochemical markers (1–7 and 7–14 days), ALP and Alizarin red staining (21 days), and mineralized matrix formation (21 days). These findings further support the notion that 10^− 6^ M insulin significantly boosts the osteogenic differentiation of human DPSCs. Interestingly, we noted no significant difference in the extracellular ALP secretion between the 10^− 6^ M insulin-treated group and the control group at days 1–7 and 7–14. ALP is a crucial enzyme involved in calcified tissue formation and extracellular matrix metabolism, serving as an early indicator of osteogenic differentiation [49]. Bakopoulou [50] previously highlighted that extensive mineral depositions hindered the penetration of the ALP substrate by MSCs from human deciduous teeth pulp, potentially explaining the comparable levels of extracellular ALP secretion in our study. Our results further reinforce the idea that insulin exerts a direct anabolic effect on human DPSCs. Nevertheless, the precise mechanism by which insulin promotes the proliferation and osteogenic differentiation of human DPSCs remains to be elucidated.

Insulin typically binds to INSR, IGF1R or INSR-IGF1R complexes on the surface of responsive cells, triggering their phosphorylation [51], and initiating various signaling pathways that impact the proliferation, differentiation, energy metabolism, and activity of osteocytes through IRS1/2 [52, 53]. In our study, we examined the gene and protein expression of receptors and receptor substrates associated with the IIS pathway. The results found that with the significant increase of cell proliferation and osteogenic differentiation, 10^− 6^ M insulin markedly suppressed the mRNA (3 and 7 days) and protein (7 days) expression of INSR, IGF1R, and IRS1. Previous research has shown that insulin treatment can induce insulin resistance in cultured cells [54–56], resulting in a reduction in cellular responsiveness to the IIS pathway and down-regulation of associated receptors and receptor substrates [57, 58]. It appears that 10^− 6^ M insulin induced insulin resistance in human DPSCs. Yang [26] confirmed that physiological insulin concentration (10^− 7^ M) initially increased INSR expression in MG-63 cells within 1 min, with a subsequent decline at 5 and 10 min, yet ultimately promoting cell proliferation and osteogenic differentiation after 72 h. In our previous study, we observed that 10^− 6^ M insulin significantly promoted INSR phosphorylation and IRS1 protein expression at 2 h in alveolar bone marrow MSCs, and obviously inhibited INSR phosphorylation and IRS1 protein expression by day 7, despite this, the treatment particularly enhanced cell proliferation and osteogenic differentiation [48]. These findings suggest that exposure to acute or chronic insulin, at either physiological or high concentrations, can result in the down-regulation of receptors and receptor substrates associated with the IIS pathway, potentially modulating the pathway’s activity. However, even under insulin resistance states, the ability of 10^− 6^ M insulin to promote DPSC proliferation and osteogenic differentiation remained consistent over time. Our results underscore that insulin has a direct anabolic effect on human DPSCs, emphasizing its role in facilitating cellular proliferation and osteogenic differentiation.

Mounting evidence suggests that the PI3K/AKT/mTOR signaling pathway, a downstream target of the IIS pathway, plays a crucial role in regulating osteoblast and osteoclast functions [59–61]. This pathway is pivotal in insulin-responsive cells and tissues [62]. Therefore, we investigated the responsiveness of human DPSCs to both the IIS and PI3K/AKT/mTOR signaling pathways under insulin resistant states and their proliferation and osteogenic differentiation capacity through inhibition experiments conducted in vitro and in vivo. Under insulin sensitive states, insulin typically initiates the PI3K cascade by phosphorylating IRS proteins, activating PI3K [63], and subsequently promoting AKT phosphorylation, thereby activating mTOR and enhancing insulin’s role in growth, proliferation, and differentiation. These proximal and distal insulin signaling events are under activation states, such as INSR, IGF1R, IRS1, PI3K, AKT, and mTOR [64]. Previous studies have shown that insulin temporarily activates IIS, and its downstream signaling pathways respond differently to insulin. For instance, Catalano [57] reported that a strong and transient phosphorylation of INSR (almost immediately upon insulin addition), ERK (at 5 min), and AKT (at 10 min) in rat hepatoma cells in response to all insulin concentrations (5 × 10^− 9^, 1.7 × 10^− 8^, 1.7 × 10^− 7^ M), followed by a very rapid decline of INRS and ERK phosphorylation at 45 min and no declines in AKT autophosphorylation even 120 min after insulin addition, the total INSR, ERK and AKT protein levels were unchanged. Yang [26] demonstrated that 10^− 7^ M insulin enhanced INSR expression in MG-63 cells at 1 min, and gradually decreased at 5 and 10 min, subsequently promoting cellular proliferation and osteogenic differentiation though the activation of MAPK and PI3K at 72 h. While Scioli [27] found that the combined platelet-rich plasma (5% v/v) and 10^− 7^ M insulin obviously inhibited IGF1R and mTOR activity in human ASCs at day 21, and IGF1R/mTOR inhibition even enhanced ASC chondro-/osteogenic differentiation. These opposite results suggest that different cells react differently to insulin downstream signal pathways under insulin resistant states, meaning that these IIS downstream signaling pathways are not necessarily chronically activated in different cells as they may be down-regulated by other modifications at other sites over time. Our inhibition experiments found that 10^− 6^ M insulin obviously down-regulated the receptors and receptor substrates protein levels associated with IIS pathway at day 7, markedly reduced the expression of the phosphorylated PI3K and the ratios of phosphorylated PI3K/total PI3K; there was no significant change in the ratios of phosphorylated AKT/total AKT; the total PI3K, phosphorylated AKT, total AKT, mTOR activity significantly increased; cellular proliferation and the protein levels of osteogenic markers significantly increased. This pattern suggests that 10^− 6^ M insulin induces insulin resistance in human DPSCs, leading to impairments in proximal insulin signaling events (INSR, IGF1R, IRS1, and PI3K) while maintaining the activity of distal pathway components (AKT and mTOR), and 10^− 6^ M insulin continues to enhance DPSC proliferation and osteogenic differentiation. In conclusion, although these studies suggest that both acute or chronic and physiological concentration or high concentration insulin can induce insulin resistance in cultured cells and gradually leads to impairment of proximal or distal insulin signaling events over time, insulin’s promotion of cell proliferation and osteogenic differentiation remains unchanged.

Notably, the inhibitor LY294002 attenuated the responsiveness of 10^− 6^ M insulin to the IIS/PI3K/AKT/mTOR pathway axis in human DPSCs, suppressing the promoting effects of insulin on proliferation and osteogenic differentiation in vitro. In vivo studies using SCID mice and rabbits with jawbone defects demonstrated that 10^− 6^ M insulin enhanced bone formation in DPSCs + nHAC constructs, showing that 10^− 6^ M insulin treatment obviously promoted DPSC proliferation and osteogenic differentiation in vitro played an important role in the in vivo bone formation of DPSCs + nHAC constructs. The inhibitor LY294002 diminished the bone formation promoted by 10^− 6^ M insulin in DPSCs + nHAC constructs, suggesting the involvement of the IIS/PI3K/AKT/mTOR pathway axis in the proliferation, osteogenic differentiation, and bone formation induced by 10^− 6^ M insulin. Overall, our results further substantiate that insulin has a direct anabolic effect on human DPSCs, emphasizing its role in promoting proliferation, osteogenic differentiation, and bone formation.

Moreover, stem cells possess the remarkable ability to exert paracrine functions, playing a pivotal role in suppressing inflammation, promoting tissue repair and regeneration, regulating immunity, and exhibiting anti-fibrotic, anti-apoptotic, and anti-oxidative effects through the secretion of cytokines [65]. Ouquerke’s research [34] highlighted that 1.5 × 10^− 2^ M insulin significantly enhanced the proliferation and osteogenic differentiation of DPSCs within 24 and 48 h by up-regulating BMPs and their receptors. Similarly, Lauritano’s findings [33] demonstrated that 1.7 × 10^− 5^ M insulin induced the proliferation and osteogenic differentiation of human DPSCs by increasing the expressions of *BMP3* and *BMP4* after 24 h and *BMP1* genes after 48 h. These studies hint at insulin’s potential to stimulate the secretion of BMPs from DPSCs, ultimately promoting cell proliferation and osteogenic differentiation by modulating the cells’ paracrine functions. In our study, we observed that 10^− 6^ M insulin effectively promoted the proliferation, osteogenic differentiation, and bone formation capacity of human DPSCs by gradually attenuating the IIS/PI3K/AKT/mTOR pathway axis under insulin resistant states. These results suggest that insulin has a direct anabolic effect on human DPSCs. Further research is needed to explore the precise mechanisms by which insulin influences the proliferation and osteogenic differentiation of human DPSCs through its functional impact on the cells, necessitating a deeper investigation into the underlying processes.

## Conclusion

In conclusion, our findings indicate that insulin induces insulin resistance in human DPSCs, resulting in impairments in proximal insulin signaling events (INSR, IGF1R, IRS1, and PI3K) while maintaining the activity of distal pathway components (AKT and mTOR). Under insulin resistant states, insulin effectively enhances the proliferation, osteogenic differentiation, and bone formation capacity of human DPSCs through gradually inducing the down-regulation of IIS/PI3K/AKT/mTOR pathway axis, showing insulin has a direct anabolic effect on human DPSCs. The application of DPSCs + nHAC + 10^− 6^ M Insulin constructs shows promise for repairing maxillofacial bone defects. However, further investigations are warranted to elucidate the specific mechanisms underlying the effects of insulin on the function of human DPSCs.

### Electronic supplementary material

Below is the link to the electronic supplementary material.

Supplementary Material 1

## Data Availability

The data that support the findings of this study are available from the corresponding author upon reasonable request.
