# Supplementary material for: Insulin promotes the bone formation capability of human dental pulp stem cells through attenuating the IIS/PI3K/AKT/mTOR pathway axis
Source: Stem Cell Res Ther. 2024 Jul 29;15:227. doi: 10.1186/s13287-024-03843-9 (PMC11287875; doi:10.1186/s13287-024-03843-9)
Supplement: Supplementary file 1 — Supplementary Material 1 [file 13287_2024_3843_MOESM1_ESM.pdf]

Supplementary Figure 1

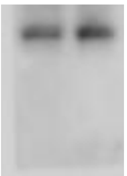

Supplementary Figure 1A (Figure 3B-COL-1)

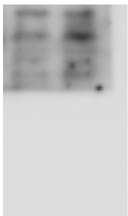

Supplementary Figure 1B (Figure 3B-ALP)

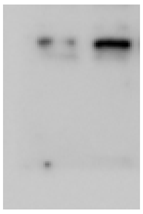

Supplementary Figure 1C (Figure 3B-OCN)

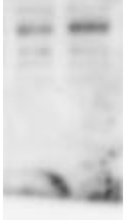

Supplementary Figure 1D (Figure 3B-RUNX2)

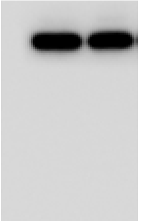

Supplementary Figure 1E (Figure 3B-GAPDH)

Supplementary Figure 2

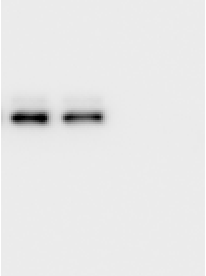

Supplementary Figure 2A (Figure 4B-INSR)

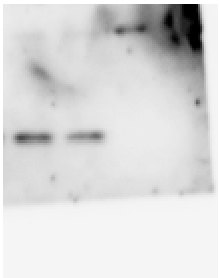

Supplementary Figure 2B (Figure 4B-IGF1R)

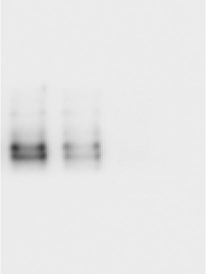

Supplementary Figure 2C (Figure 4B-IRS1)

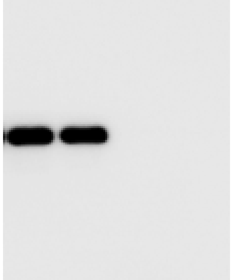

Supplementary Figure 2D (Figure 4B-GAPDH)

Supplementary Figure 3

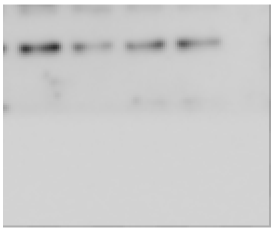

Supplementary Figure 3A (Figure 5A-INSR)

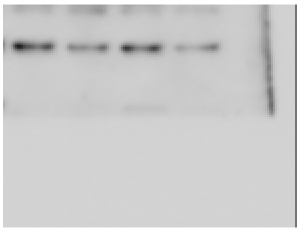

Supplementary Figure 3B (Figure 5A-IGF1R)

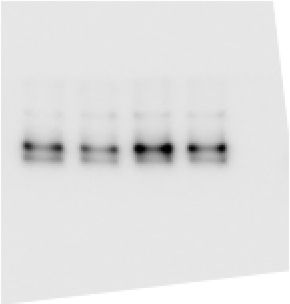

Supplementary Figure 3C (Figure 5A-IRS1)

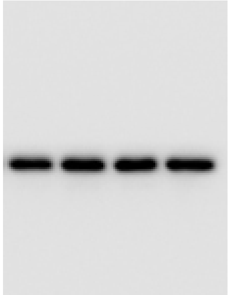

Supplementary Figure 3D (Figure 5A-GAPDH)

Supplementary Figure 4

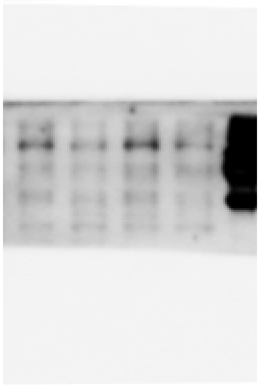

Supplementary Figure 4A (Figure 5B-p-PI3K)

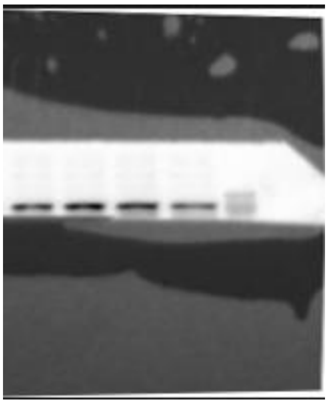

Supplementary Figure 4B (Figure 5B-PI3K)

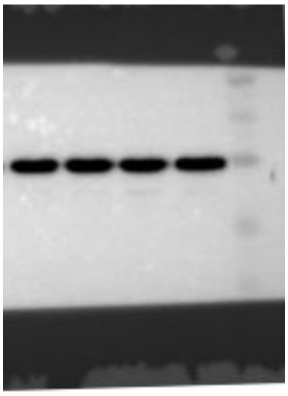

Supplementary Figure 4C (Figure 5B-GAPDH)

Supplementary Figure 5

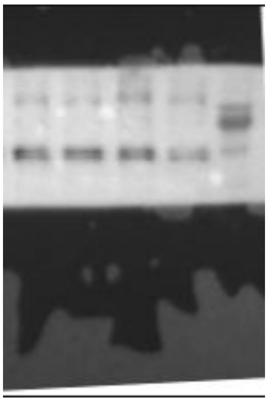

Supplementary Figure 5A (Figure 5C-p-AKT)

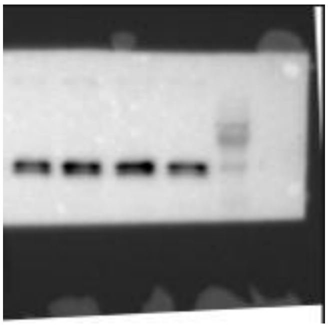

Supplementary Figure 5B (Figure 5C-AKT)

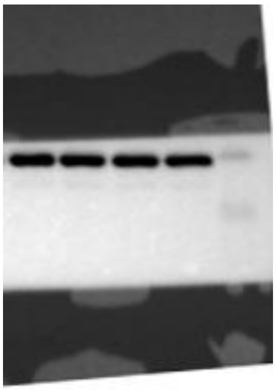

Supplementary Figure 5C (Figure 5C-GAPDH)

Supplementary Figure 6

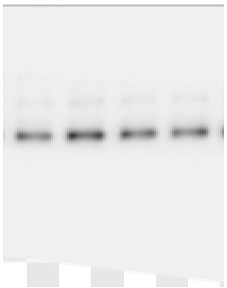

Supplementary Figure 6A (Figure 5D-mTOR)

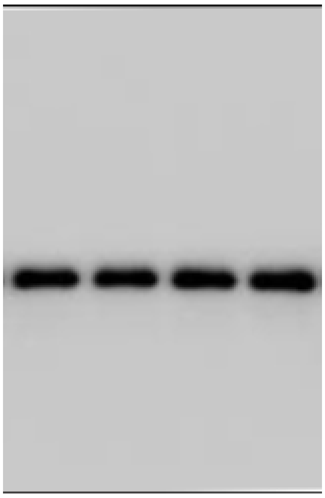

Supplementary Figure 6B (Figure 5D-GAPDH)

Supplementary Figure 7

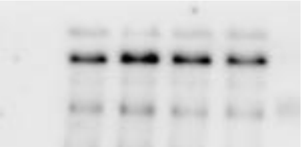

Supplementary Figure 7A (Figure 6B-COL-1)

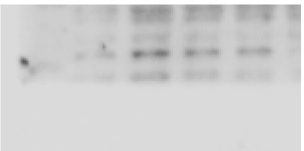

Supplementary Figure 7B (Figure 6B-ALP)

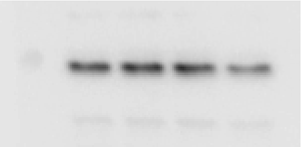

Supplementary Figure 7C (Figure 6B-OCN)

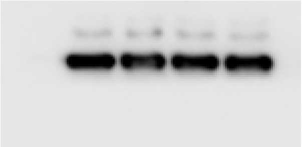

Supplementary Figure 7D (Figure 6B-GAPDH)

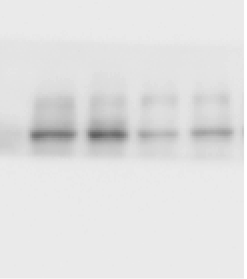

Supplementary Figure 7E (Figure 6B-RUNX2)

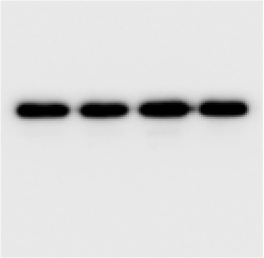

Supplementary Figure 7F(Figure 6B-GAPDH)
